# Oral High-Dose Atorvastatin Treatment in Relapsing-Remitting Multiple Sclerosis

**DOI:** 10.1371/journal.pone.0001928

**Published:** 2008-04-09

**Authors:** Friedemann Paul, Sonia Waiczies, Jens Wuerfel, Judith Bellmann-Strobl, Jan Dörr, Helmar Waiczies, Mareile Haertle, Klaus D. Wernecke, Hans-Dieter Volk, Orhan Aktas, Frauke Zipp

**Affiliations:** 1 Cecilie Vogt Clinic for Neurology in the HELIOS Clinic Berlin-Buch, Charité – University Medicine Berlin and Max-Delbrueck Center for Molecular Medicine, Berlin, Germany; 2 Department of Neurology in the HELIOS Clinic Berlin-Buch, Berlin, Germany; 3 Sostana, Department of Medical Statistics, Berlin, Germany; 4 Institute of Immunology, Charité – University Medicine Berlin, Berlin, Germany; National Institute of Neurological Disorders and Stroke, United States of America

## Abstract

**Background:**

Recent data from animal models of multiple sclerosis (MS) and from a pilot study indicated a possible beneficial impact of statins on MS.

**Methodology/Principal Findings:**

Safety, tolerability and effects on disease activity of atorvastatin given alone or in combination with interferon-beta (IFN-β) were assessed in a phase II open-label baseline-to-treatment trial in relapsing-remitting MS (RRMS). Patients with at least one gadolinium-enhancing lesion (CEL) at screening by magnetic resonance imaging (MRI) were eligible for the study. After a baseline period of 3 monthly MRI scans (months −2 to 0), patients followed a 9-month treatment period on 80 mg atorvastatin daily. The number of CEL in treatment months 6 to 9 compared to baseline served as the primary endpoint. Other MRI-based parameters as well as changes in clinical scores and immune responses served as secondary endpoints. Of 80 RRMS patients screened, 41 were included, among them 16 with IFN-β comedication. The high dose of 80 mg atorvastatin was well tolerated in the majority of patients, regardless of IFN-β comedication. Atorvastatin treatment led to a substantial reduction in the number and volume of CEL in two-sided multivariate analysis (p = 0.003 and p = 0.008). A trend towards a significant decrease in number and volume of CEL was also detected in patients with IFN-β comedication (p = 0.060 and p = 0.062), in contrast to patients without IFN-β comedication (p = 0.170 and p = 0.140). Immunological investigations showed no suppression in T cell response but a significant increase in IL-10 production.

**Conclusions/Significance:**

Our data suggest that high-dose atorvastatin treatment in RRMS is safe and well tolerated. Moreover, MRI analysis indicates a possible beneficial effect of atorvastatin, alone or in combination with IFN-β, on the development of new CEL. Thus, our findings provide a rationale for phase II/III trials, including combination of atorvastatin with already approved immunomodulatory therapy regimens.

**Trial Registration:**

ClinicalTrials.gov NCT00616187

## Introduction

Multiple sclerosis (MS) is a chronic inflammatory demyelinating disease of the central nervous system causing pronounced neurological disability in younger adults. Although incurable, several disease-modifying drugs (DMD) such as beta-interferons (IFN-β), glatiramer acetate, mitoxantrone and recently natalizumab have proven to be effective in reducing the number of relapses. However, a beneficial influence of DMD on the progression of disability is far less pronounced and still a matter of debate. A substantial number of patients do not respond to current DMD, or refuse long-term adherence to these drugs due to intolerable side-effects or the inconvenience of parenteral application. Therefore, the development of oral DMD alternatives has stimulated scientific research and encouraged clinical trials. Nevertheless, no orally applicable first line drug has reached approval for the treatment of relapsing-remitting MS (RRMS) to date.

Statins are orally administered cholesterol-lowering agents established in the treatment of cardiovascular diseases [1]. Recently, the presumed immunomodulatory and potential neuroprotective effects of these 3-hydroxy-3-methylglutaryl (HMG)-CoA reductase inhibitors have attracted increasing interest [2]. Indeed, oral statins were effective in preventing and reversing relapsing paralysis in experimental autoimmune encephalomyelitis (EAE), an animal model of MS [3]–[5]. A previous pilot study with oral simvastatin given daily over 6 months showed a significant reduction of contrast-enhancing lesions (CEL) in brain magnetic resonance imaging (MRI) of 30 RRMS patients compared to a 3 month baseline period [6]. Atorvastatin, which powerfully suppresses T cell activation and inducible MHC class II expression on antigen-presenting cells *in vitro* and *in vivo*, was not only superior to other statins in these immunological properties [7], but also apparently had beneficial effects in a randomized placebo-controlled treatment trial for rheumatoid arthritis at a daily dose of 40 mg [8]. These data suggest the potential value of statins in the treatment of MS. Thus, we investigated the safety, tolerability and therapeutic potential of high-dose oral atorvastatin (80 mg daily) given alone or in combination with beta-interferons, and here report the results of a phase II open-label baseline-to-treatment trial in a cohort of 41 RRMS patients.

## Methods

### Study design and participants

A baseline-to-treatment trial was designed to evaluate the safety, tolerability and efficacy of orally administered atorvastatin in patients with RRMS. Patients were screened and enrolled in the outpatient clinic of the Cecilie Vogt Clinic at the Charité – University Medicine Berlin. The protocol for this trial and supporting CONSORT checklist are available as supporting information; see Checklist S1 and Protocol S1. The protocol was reviewed and approved by the ethics committee of the Charité, and the German Federal Institute for Drugs and Medical Devices (BfArM) was notified regarding the initiation of the trial. The study was supervised by an independent data monitoring board. Before providing informed written consent, all patients were advised of the approved alternative therapies available to them. Staff members performing the magnetic resonance imaging (MRI) were blinded for the clinical course, and physicians assessing the neurological status of the patients were blinded for the MRI results.

Sample size calculation was based on an analysis of variance for repeated measures, and determined with α = 5% (two-sided), power = 80%, a between-level correlation of 0.3, and supposing 2.31±1.39 gadolinium (Gd-DTPA)-enhancing lesions (CEL) before and 1.30±0.99 CEL after treatment [6]. Using nQuery Advisor 5.0 (Statistical Solutions, Cork, Ireland), we calculated a sample size of n = 34, resulting in a total sample size of n = 41 with a drop-out rate of 20%. The study population consisted of RRMS outpatients who fulfilled the panel criteria for clinically definite MS [9] with an Expanded Disability Status Scale (EDSS) between 0 and 6, age 18–55, and with at least one CEL on a qualifying T1-weighted brain MRI scan. Clinically active disease at the time of screening, i.e. symptoms of a relapse, was not a prerequisite for inclusion. Patients had either not received any DMD for at least 6 months prior to screening (n = 25), or had received a DMD with either IFN-β-1a 22 µg s.c. 3 times weekly (n = 9) or IFN-β-1b s.c. every other day (n = 7) for at least 6 months. In the DMD group, IFN-β treatment was continued throughout the entire study. Following the qualifying MRI, performed to demonstrate disease activity (visit −3), the individual study period lasted 12 months, with a baseline phase of 3 monthly MRI scans and a 9-month treatment phase. Each patient made 13 regular visits to our outpatient clinic, with monthly MRI examinations, Multiple Sclerosis Functional Composite (MSFC) performance, and EDSS rating conducted every 3 months (at visits −3, 0, 3, 6, 9, 12). After 3 baseline visits, patients received 80 mg atorvastatin (40 mg twice daily) during the 9-month treatment period. Treatment of relapses was performed according to current guidelines, with 1 g methylprednisolone (MP) administered intravenously for 3 to 5 days. Atorvastatin treatment was continued during relapse treatment. However, in the case of MP administration, subsequent MRI examination was postponed to ensure an interval of 4 weeks after the last day of MP application, so as to avoid confounding effects of corticosteroid treatment on MRI contrast enhancement [10]. The same interval was adhered to with regards to relapses requiring MP treatment prior to the screening MRI.

### Efficacy endpoints

The primary endpoint was the number of CEL at months 6 to 9 of treatment compared to baseline (months −2 to 0). Secondary MRI outcome variables included the volume of CEL, number and volume of hyperintense lesions on T2-weighted scans (“T2-lesion load”), volume of T1-hypointense lesions (“black hole” = BH), whole brain magnetization transfer ratio (MTR), and apparent diffusion coefficient (ADC) of the normal appearing white matter (NAWM) at months 6 to 9 compared to baseline. Further secondary endpoints were changes in EDSS and MSFC scores. Other planned targets were changes in various peripheral immune cell parameters from the baseline period to the treatment phase (months 6 and 9).

### Safety and tolerability

Safety and tolerability of the study drug were assessed by monthly MRI scans, physical and neurological examination, relapse assessment, electrocardiogram and vital signs (blood pressure, pulse). Laboratory examinations (performed at visits −3, 0, 1, 3, 6 and 9) recorded red and white blood counts, liver enzymes (ALT, AST, γ-GT), electrolytes (sodium, potassium, chloride), creatinine and creatine kinase (CK). At months 0 and 9, total cholesterol, HDL and LDL cholesterol, and triglycerides were investigated.

### Procedures

#### Magnetic resonance imaging

MRI measurements were performed on a scanner operating at 1.5 T (Siemens Sonata, Siemens Medical Systems, Erlangen, Germany). A triple echo spin-echo sequence (TR 5,780 ms, TE_1_ 13 ms, TE_2_ 81 ms, TE_3_ 121 ms, 3 mm slice thickness and 44 contiguous axial slices) was used to obtain proton density and T2-weighted images. Additionally, we applied a fluid-attenuated inversion recovery sequence (TIRM, TR 10,000 ms, TE 108 ms, TI 2,500 ms, 3 mm slice thickness and 44 contiguous axial slices) and a high resolution 3-dimensional T1-weighted sequence (MPRAGE, TR 2,110 ms, TE 4.38 ms, TI 1,100 ms, flip angle 15°, resolution 1 mm^3^). Conventional spin-echo T1-weighted (TR 1,060 ms, TE 14 ms, 3 mm slice thickness and 44 contiguous axial slices) and magnetization-prepared images (MTI, TR 1,290 ms, TE 14 ms, 3 mm slice thickness and 44 contiguous axial slices) were obtained before and 5 minutes after injection of 0.1 mmol/kg Gd-DTPA (Magnevist, Bayer-Schering, Berlin, Germany). An epi-planar (EPI) diffusion-weighted sequence (DWI, TR 9,400 ms, TE 118 ms, 3 mm slice thickness, matrix 128×128, b values 1 and 1,000 s/mm^2^) was acquired in 3 directions for the calculation of the ADC. A series of axial, coronal and sagittal images was obtained to create a reference scan for subsequent accurate repositioning of patients at follow-up. The axial slices were positioned to run parallel to a line that joined the most inferior-anterior and inferior-posterior parts of the corpus callosum. Image quality was reviewed according to pre-determined criteria.

Raw data were transferred to a Linux workstation and processed following a semi-automated procedure described previously [11], including an image coregistration (FMRIB's Linear Image Registration Tool, FMRIB Analysis Group, University of Oxford, Oxford, UK) and inhomogeneity correction routine embedded into the MedX v.3.4.3 software package (Sensor Systems Inc., Sterling, VA, USA). Bulk white matter lesion load and lesion count of T2-weighted scans, as well as number and volume of CEL and hypointense lesions on T1-weighted scans, were routinely measured using the MedX v.3.4.3 software package. MTR was calculated in MIPAV (Medical Image Processing, Analysis, and Visualization, CIT-NIH, Bethesda, MD, USA) as previously described [12]. MRI analyses were conducted in an anonymized way, applying a semi-automated procedure. Experienced raters (JW, HW and MH) were blinded to clinical data and time of investigation.

#### Immunological examinations

In light of the reported anti-proliferative and anti-inflammatory properties of statins and IFN-β [13], we performed *in vitro* assays to test the synergy of these agents. T cell proliferation and gene expression of the tumor necrosis factor (TNF)-related apoptosis-inducing ligand (TRAIL) were chosen as a response marker for IFN-β treatment [14]. The proliferation of myelin basic protein (MBP)-specific T cell lines towards anti-CD3/anti-CD28 was measured in the presence of increasing doses of atorvastatin (1 nM–50 µM) and IFN-β-1a (0.0001–100,000 IU/ml) by a standard ^3^H thymidine incorporation assay. *TRAIL* expression was measured in peripheral blood mononuclear cells (PBMC) treated with atorvastatin (100 nM–1 µM) and IFN- β-1a (1–10,000 IU/ml) for 4 h using real-time quantitative reverse-transcriptase PCR (rtPCR), and is reported as relative gene expression normalized to the housekeeping gene GAPDH, as previously described [14]. Drug interactions were investigated using isobolographic analyses as previously described [15].

In patients, we concentrated on proliferative responses and expression of key cytokines. All immunological measurements were performed by independent investigators who were unaware of the clinical and MRI data. For the analysis of proliferative responses, PBMC were isolated from patients' whole blood using standardized protocols, and plated on freshly thawed 96-well culture plates containing increasing doses of a recall antigen cocktail consisting of the following: CMV viral lysate diluted at 1∶1,000 (ABI, Columbia, MA, USA); *C. albicans* (Candidin, Allergopharma, Reinbek, Germany) at a final concentration of 10 µl/ml; purified tuberculin (PPD, Chiron Behring, Liederbach, Germany) at a final dilution of 1∶250; tetanus toxoid (Tetasorbat SSW, SmithKline Beecham Pharma, Munich, Germany) at 1∶1,000; and influenza antigen vaccine 2002 (Aventis Pasteur, Lyon, France) at 1∶1,000. ConA (Sigma, Munich, Germany) was used as an antigen-independent activator. Proliferation was measured in counts per minute (cpm) by a standard ^3^H thymidine incorporation assay, and maximum proliferative response was calculated on an index between the unstimulated control and the maximum proliferation counts. Soluble interleukin (IL)-4, IL-10, TNF-α and IFN-γ were measured after 24 h ConA stimulation by cytometric multiplexing with the BD™ Cytometric Bead Array (CBA), following the manufacturer's instructions (Becton Dickinson Biosciences, Heidelberg, Germany). *TRAIL* expression was measured by rtPCR from whole blood collected in PAXgene™ Blood RNA Tubes (PreAnalytiX, Hombrechtikon, Switzerland). Blood was processed using the PAXgene™ Blood RNA Kit, following the manufacturer's instructions. Reverse transcription, amplification and design of *Taq*Man primer and probes were performed as previously described [14].

#### Statistical analysis

As required by the inclusion criteria, brain MRI at enrolment showed disease activity with respect to the occurrence of CEL. However, to achieve a stable baseline as a prerequisite for adequate statistical testing, and thus to avoid any possible statistical bias, we excluded data from this first MRI from further statistical analysis and defined months −2 to 0 as the baseline period. Baseline was compared to the treatment period months 6 to 9, following the hypothesis that presumed effects of atorvastatin on inflammatory disease activity would be detectable after at least 6 months of treatment [6], [8]. Therefore, statistical analyses were carried out only in patients who had completed at least 6 months of atorvastatin treatment. For the analyses, we used nonparametric multivariate analysis of variance (MANOVA) for repeated measurements, which allowed all 4 treatment time points and all 3 baseline measurements to be analyzed simultaneously with respect to time course [16]. For exploratory comparison of the two subgroups of patients with or without IFN-β comedication, this analysis was also carried out in a two-factorial design. The Wilcoxon signed ranks test was used to examine changes in routine laboratory parameters between the baseline and treatment periods and the annualized relapse rate before and during atorvastatin treatment. Significance was assessed at the p<0.05 level. Regarding the primary endpoint of CEL number, this p-level is to be understood in a confirmatory sense. P-values of secondary endpoints and in subgroup analyses are, however, to be understood in an exploratory sense and adjustments for multiple comparisons were therefore not carried out. All numerical calculations were performed using SPSS 13 (SPSS Inc., Chicago, IL, USA) and SAS 9.1 (SAS Inc., Cary, NC, USA).

## Results

### Primary and secondary endpoints

**Figure 1:**
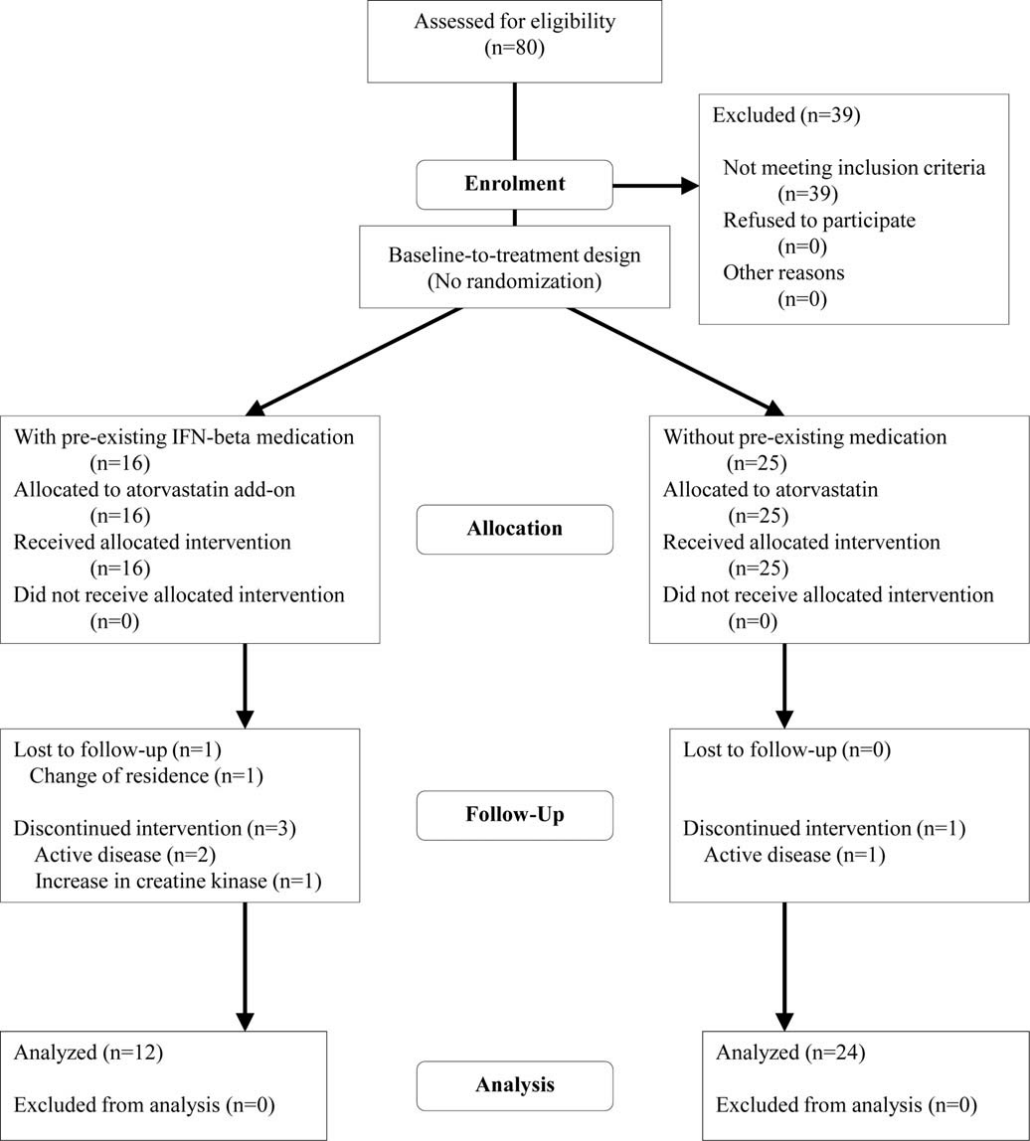
Flow chart of study patients.

Of the 80 patients screened, 41 were subsequently enrolled in the trial (Figure 1). The clinical features of these patients are summarized in Table 1. Thirty-nine patients were excluded because they did not show a CEL on the qualifying MRI scan. Five patients discontinued the study before completion of at least 6 months of atorvastatin treatment. Thus, data from 36 patients were available for the final analysis of the primary endpoint. Treatment with high-dose atorvastatin resulted in a significant reduction of CEL number compared to baseline in the multivariate analysis (p = 0.003, Table 2). Concerning the two subgroups, we found a trend towards a significant CEL number reduction over time in patients with IFN-β comedication (p = 0.060), but not in patients without IFN-β comedication (p = 0.170, Table 2). Direct exploratory comparison of these two groups using a two-factorial MANOVA design showed no differences (p = 0.274).

**Table 1:** Clinical and demographic baseline data of patients.

	Sex (m, f)	Age (yrs)	Duration of disease in months	Duration of IFN-β pre-treatment in months	EDSS at inclusion	Total no. of relapses since disease onset	No. of relapses 12 months prior to treatment
Total cohort (n = 41)	20/21	35.4 (19–51)	84 (2–317)	n.a.	1.67 (1.4, 0–6)	4.07 (2.6, 1–12)	1.46 (1.1, 0–4)
w/IFN-β (n = 16)	9/7	37.9 (24–48)	116.3 (26–317)	48 (7–115)	2.50 (1.5, 0–6)	5.75 (2.6, 3–12)	1.63 (1.3, 0–4)
w/o IFN-β (n = 25)	11/14	33.9 (19–51)	63.4 (2–229)	n.a.	1.14 (1.1, 0–4)	3 (2.1, 1–10)	1.4 (1.0, 0–4)

Values are mean (standard deviation, range). Abbreviations: w, w/o IFN-β: with/without interferon-beta pre-treatment/comedication.

**Table 2:** Primary and secondary MRI and clinical endpoints in the baseline and treatment periods (mean, standard deviation, range).

	Baseline (mo −2/0), mean, SD, range			Treatment (mo +6/+9), mean, SD, range			P (MANOVA)		
	All analyzed (36)	w/o IFN-β (24)	w/IFN-β (12)	All analyzed (36)	w/o IFN-β (24)	w/IFN-β (12)	All	w/o IFN-β	w/IFN-β
CEL (no)	2 (2.6) (0–13.3)	2.26 (3.0) (0–13.3)	1.47 (1.41) (0.3–4.7)	1.52 (2.18) (0–8.75)	1.88 (2.58) (0–8.75)	0.81 (0.62) (0–1.75)	0.003	0.170	0.060
CEL vol (mm^3^)	120 (170) (0–672)	141 (197) (0–672)	79 (94) (4–336)	106 (177) (0–806)	133 (209) (0–806)	50 (55) (0–148)	0.008	0.140	0.062
T2 lesion count (no)	45.6 (28.9) (2–136)	41.6 (26.5) (2–111)	53.6 (33) (22–136)	49.4 (29.5) (2–139)	45 (27.4) (2–120)	58 (32.8) (24–139)	<0.001	0.002	0.008
T2 lesion vol (mm^3^)	5142 (4065) (217–20766)	4155 (2881) (217–11268)	7116 (5370) (2077–20766)	5797 (4566) (79–24097)	4757 (3251) (79–12255)	7879 (6096) (2336–24097)	0.008	0.053	0.003
BH vol (mm^3^)	331 (544) (0–2286)	211 (389) (0–1356)	569 (731) (0–2286)	338 (571) (0–2476)	211 (377) (0–1378)	596 (796) (0–2476)	0.560	0.321	0.688
MTR Whole brain	0.197 (0.011) (0.170–0.225)	0.199 (0.011) (0.179–0.225)	0.191 (0.01) (0.170–0.206)	0.197 (0.011) (0.171–0.214)	0.198 (0.01) (0.173–0.212)	0.196 (0.013) (0.171–0.214)	0.350	0.331	0.023
ADC NAWM	73.7 (5) (68.3–89.9)	73.4 (5.3) (68.3–89.9)	74.3 (4.6) (69.3–84.8)	73.3 (4.8) (67.4–88)	73.1 (4.8) (67.5–88)	73.8 (5.1) (67.4–85.8)	0.108	0.066	0.656
EDSS	1.58 (1.35)§ (0–6)	1.19 (1)§ (0–4)	2.38 (1.63)§ (0–6)	1.57 (1.32) (0–6)	1.16 (0.93) (0–4)	2.4 (1.62) (0–6)	0.665	0.502	0.712
MSFC	0.2 (0.69) (−2.35–1.07)	0.42 (0.47) (−0.53–1.07)	−0.23 (0.86) (−2.35–0.52)	0.46 (0.63) (−2.14–1.19)	0.65 (0.42) (−0.15–1.19)	0.071 (0.81) (−2.14–1.04)	<0.001	<0.001	0.035

P-values are calculated by means of multivariate analysis (MANOVA).

§ month 0. Abbreviations: SD: standard deviation; w, w/o IFN-β: with/without interferon-beta pre-treatment/comedication; CEL: contrast enhancing lesions; BH: black hole; MTR: magnetization transfer ratio; ADC: apparent diffusion coefficient; NAWM: normal appearing white matter; EDSS: Expanded Disability Status Scale; MSFC: Multiple Sclerosis Functional Composite.

Concerning the CEL volume as a secondary endpoint, we found a similar pattern: multivariate analysis revealed a significant reduction in CEL volume compared to baseline in the analyzable population as a whole (p = 0.008). In the subgroup with IFN-β comedication, there was a trend towards a significant reduction of CEL volume (p = 0.062) which was not the case in the group without IFN-β comedication (p = 0.140, Table 2). Again, no differences between groups with and without IFN-β were observed in the direct two-factorial MANOVA comparison (p = 0.315). Number and volume of T2 lesions increased in both the entire study population and the two subgroups over time without relevant inter-group differences. Whole brain MTR increased significantly during our study only in the IFN-β comedication group, but not in patients without IFN-β comedication (Table 2). Black hole evolution and NAWM ADC did not change over time either in the entire study population or in the subgroups (Table 2). EDSS scores remained unchanged over the entire study period (p = 0.665, Table 2). Better MSFC scores (Table 2) resulted from improvements in the PASAT (Paced Auditorial Serial Addition Test) and the 9-HPT (9-Hole Peg Test) subtests, while the TWT (Timed Walk Test) remained unchanged (data not shown). In the study population as a whole, the mean annualized relapse rate ± standard deviation (SD) was 1.46±1.1 in the year prior to atorvastatin treatment and 0.68±0.99 during atorvastatin treatment (p<0.001). In the subgroup of patients without IFN-β comedication the relapse rate was 1.4±1.0 in the year before treatment and 0.64±1.0 during treatment (p = 0.002). In the IFN-β comedication group the relapse rate was 1.63±1.26 before treatment and 0.75±0.97 during treatment (p = 0.049).

Concerning immunological effects, our *in vitro* studies indicated synergistic activity of atorvastatin and IFN-β, which was the basis for introducing the combination therapy arm in the clinical trial. Using MBP-specific T cell lines we performed proliferation assays to measure inhibition in T cell response following increasing doses of both drugs. The EC_50_ (concentration producing 50% of maximum proliferation inhibition) for atorvastatin was 3.2 µM and for IFN-β 270 IU/ml. From these two EC_50_ values we were able to construct an isobologram and theoretically determine combinations of low concentrations of both drugs that would potentially have a supra-additive effect (synergism) (see Methods). The lowest concentration combination tested, which in fact revealed EC_50_ in culture and fell within the synergy zone of the isobologram, was IFN-β, atorvastatin: 100 IU/ml, 0.1 µM. For the expression of *TRAIL*, which our previous work had suggested to be a response marker for IFN-β therapy in MS [14], the EC_50_ of atorvastatin was 0.6 µM, and for IFN-β 900 IU/ml. The lowest concentrations exhibiting synergy were IFN-β, atorvastatin: 100 IU/ml, 0.1 µM.

Applying 80 mg atorvastatin daily *in vivo*, however, revealed neither an overall antiproliferative effect on peripheral T cells (mean stimulation index ± SD upon recall antigen challenge at baseline: 112.7±124.6, on treatment: 92.7±98.7, p = 0.26; mean stimulation index ± SD upon ConA challenge at baseline: 83.1±82.8, on treatment: 85.4±79.8, p = 0.66) nor an effect on basal *TRAIL* levels (mean relative gene expression ± SD at baseline: 15.5±12.3, on treatment: 20.9±20.8, p = 0.23). Furthermore, while IL-4 levels did not change over time (mean IL-4 level ± SD at baseline: 86.4 pg/ml±48.1, on treatment: 92.4 pg/ml±53.5, p = 0.47), we observed a significant increase in the regulatory cytokine IL-10 (mean IL-10 level ± SD at baseline: 202.3 pg/ml±99.0, on treatment: 256.4 pg/ml±140.1, p = 0.02). We observed a borderline increase for TNF-α levels (mean level ± SD at baseline: 1,661.3 pg/ml±807.0, on treatment: 1,914.3 pg/ml±1,132.8, p = 0.05), while IFN-γ levels remained unchanged (mean level ± SD at baseline: 9,148.1 pg/ml±6,598.0, on treatment: 10,899.4 pg/ml±11,208.1, p = 0.42).

### Treatment adherence and tolerability

Serum levels of total cholesterol, LDL cholesterol and triglycerides significantly decreased from baseline until the end of the study, while HDL cholesterol remained unchanged, indicating patient compliance and pharmacological effects of atorvastatin in our cohort (Table 3). Of the 41 patients enrolled in this study, 5 patients discontinued before completion of 6 months of atorvastatin treatment (Table 4), 3 of them due to a severe relapse (pat. #1 and pat. #3 with brainstem symptoms; pat. #2 with gait disturbance). In the remaining evaluable study population (n = 36), 15 participants (13 without IFN-β comedication, 2 with IFN-β comedication) were treated per protocol, i.e. these patients received 80 mg atorvastatin daily throughout the entire study (PP group); in 14 patients (6 without IFN-β comedication, 8 with IFN-β comedication) minor protocol violations (MPV) occurred, e.g. these patients received a mean daily dose (MDD) of at least 70 mg atorvastatin throughout the study (MDD 76.2 mg, MPV group); 7 patients (5 without IFN-β comedication, 2 with IFN-β comedication) caused major protocol violations (MAJ) by incorporating a mean daily dose of less than 70 mg atorvastatin (MDD 48.4 mg, MAJ group) (Table 5). The high dose of 80 mg atorvastatin was well tolerated in the majority of patients, regardless of IFN-β comedication. 16 patients experienced a temporary mild (less than 1.5-fold the upper limit) elevation of liver enzymes with no consistent timeframe of occurrence after the initiation of atorvastatin treatment. In 5 subjects a clinically relevant elevation of transaminases was detected (up to 4-fold the upper limit), though after temporary discontinuation of the study drug or dose reduction these parameters returned to normal. In 10 patients (24.4%), an elevation of serum CK was observed of less than 1.5-fold the upper limit in 6 cases and more than 1.5-fold in 4 patients, resulting in a dose reduction or withdrawal from the study (see details below on protocol violations, and Tables 4 and 6). However, no cases of myoglobinuria and/or rhabdomyolysis occurred. Further side effects are listed in Table 6. Reasons for protocol violations were as follows: 11 patients from the MPV group had a short run-in phase of 1 or 2 weeks to improve tolerability of atorvastatin (start with 20 mg twice daily). This minor protocol violation was initiated during the course of the study, following up several patients who experienced reduced tolerability and side effects (nausea, diarrhoea) when immediately starting on 80 mg daily. One patient had a temporary dose reduction to 40 mg daily owing to increase of AST more than 2-fold the upper limit. A second patient had a temporary dose reduction to 40 mg due to an elevation of CK 1.5-fold the upper limit, and a third patient temporarily reduced the dose because of nausea and dizziness under the original dosage. Reasons for substantial dose reduction in the MAJ group were as follows: 4 patients temporarily discontinued or reduced the daily atorvastatin dose to 20 mg or 40 mg because of an elevation of liver enzymes up to 4-fold the upper limit, though without clinical signs of hepatic dysfunction; one patient experienced an elevation of CK 2.5-fold the upper limit, complaining of diffuse muscle pain; another patient experienced recurrent diarrhoea and later developed a lumbar herpes zoster on 80 mg atorvastatin; a further patient withdrew after 7 months of treatment owing to a 5-fold increase in CK and intolerable myalgias. One additional patient withdrew prematurely owing to a 10-fold increase in CK (pat. #4, Table 4).

**Table 3:** Change of cholesterol and triglyceride serum levels under treatment with atorvastatin in the evaluable study population (n = 36). SD: standard deviation.

	Before treatment (mo 0) (mean in mg/dl, SD, range)	End of treatment (mo +9) (mean in mg/dl, SD, range)	P (Wilcoxon Signed Ranks Test)
Cholesterol			
- Total	180.1 (33.3, 109–292)	123.5 (23.6, 75–200)	<0.001
- LDL	104.9 (29.6, 36–191)	51.2 (17.3, 13–106)	<0.001
- HDL	58.6 (14.1, 29–87)	60.9 (14.1, 26–94)	0.231
Triglycerides	113.1 (56, 42–249)	92.7 (53.2, 28–264)	0.002

**Table 4:** Clinical characteristics and MRI data of patients with premature study withdrawal.

Pat.	Sex, age	IFN-β treatment	WD mo.	CEL (no) BL	CEL (no) Tx	CEL (vol) BL	CEL (vol) Tx	Reason for WD, comments
#1	M, 39	IFN-β-1a 22 µg	+4	0.66	2.5	98.25	193.8	Relapse, active disease: 2 relapses in 12 mo prior to inclusion, dose augmentation to IFN-β-1a 44 µg
#2	M, 44	IFN-β-1a 22 µg	+1	0	0	0	0	Relapse, preceding relapse during BL, switch to IFN-β-1a 44 µg and shortly thereafter mitoxantrone
#3	M, 27	None	+4	6	3.5	423.5	171.7	Relapse, active disease: 2 relapses in 12 mo prior to inclusion, 2 relapses in BL, started IFN-β-1a 44 µg
#4	M, 33	IFN-β-1b	+5	0.33	0	21.5	0	10-fold increase in creatine kinase, no relapse or progression of disability during treatment period
#5	M, 24	IFN-β-1b	+5	0	0	0	0	Change of residence, no relapse or progression of disability during treatment period

For CEL, mean values of BL (baseline) and treatment period (Tx) until discontinuation are given. Abbreviations: WD: withdrawal; CEL: contrast enhancing lesions.

**Table 5:** Number and percentage of patients treated per protocol (PP; 80 mg atorvastatin daily), with minor protocol violations (MPV; mean daily dose (MDD) at least 70 mg atorvastatin daily), with major protocol violations (MAJ; mean daily dose less than 70 mg atorvastatin daily) or with premature withdrawal before completing 6 months of treatment (PW).

	PP (MDD 80 mg)	MPV (MDD 76.2 mg)	MAJ (MDD 48.4 mg)	PW
Total cohort (n = 41)	15 (36.6%)	14 (34.1%)	7 (17.1%)	5 (12.2%)
w/IFN-β (n = 16)	2 (12.5%)	8 (50%)	2 (12.5)	4 (25)
w/o IFN-β (n = 25)	13 (52%)	6 (24%)	5 (20%)	1 (4%)

**Table 6:** Frequency of adverse events as a percentage of study participants (n = 41).

Adverse event	Total cohort (n)	Total cohort (%)	w/IFN-β (n)	w/IFN-β (%)	w/o IFN-β (n)	w/o IFN-β (%)
Respiratory tract infections^1^	23	56.1	9	56.3	14	56
Elevated liver enzymes	21	51.2	10	62.5	11	44
Elevated creatine kinase	10	24.4	7	43.8	3	12
Pain (back, chest, joints)	10	24.4	4	25	6	24
Laboratory abnormalities^2^	17	41.5	9	56.3	8	32
Myalgia	7	17.1	2	12.5	5	20
Headache	6	14.6	2	12.5	4	16
Fatigue	6	14.6	1	6.3	5	20
Diarrhoea	6	14.6	2	12.5	4	16
Genital infections	4	9.8	2	12.5	2	8
Other infections	4	9.8	3	18.8	1	4
Nausea, flatulence	4	9.8	3	18.8	1	4
Abdominal pain	4	9.8	0	0	4	16
Herpes labialis/Herpes zoster	3	7.3	0	0	3	12

1 Rhinitis, sinusitis, bronchitis.

2 Clinical insignificant deviations from the normal range (blood count, electrolytes).

## Discussion

Our study – the longest statin treatment in MS patients reported thus far, and the first clinical trial examining a combination treatment of atorvastatin with IFN-β – suggests that treatment with high-dose atorvastatin over a period of 9 months is safe and well tolerated in the majority of patients. Moreover, we observed a pronounced reduction in number and volume of CEL under treatment when compared to baseline. These results are consistent with, and go beyond, those of a recent report describing the effects of 80 mg simvastatin given to 30 patients with RRMS over a period of 6 months [6]. Regarding primary endpoints, our data are comparable to this study as we observed (i) a decrease in mean CEL number from 2 to 1.52 (as compared to a decrease from 2.31 to 1.30 reported by Vollmer et al. [6]), and (ii) a reduction of CEL volume from 120 mm^3^ to 106 mm^3^ (as compared to a decrease from 234 mm^3^ to 139 mm^3^ reported by Vollmer et al.). However, in the latter study, mean T2 lesion volume at baseline was considerably higher than in our cohort (27019 mm^3^ vs. 5142 mm^3^) and, in contrast to our results, did not increase further. This may reflect the difference in clinical baseline parameters between the two study populations occurring as a result of lower mean age (35 vs. 44 yrs) and a lower EDSS at study entry (1.7 vs. 2.8) in our patient group.

The baseline-to-treatment study design used here may be subject to certain probabilistic phenomena such as regression to the mean, i.e. an expected decrease of disease activity in a given patient population with high disease activity at study onset. However, by applying 3 monthly MRI scans prior to treatment initiation, thus resulting in a stable baseline as a prerequisite for adequate statistical testing, and by performing a nonparametric MANOVA, we used all means possible to avoid statistical bias. These evaluation methods also clearly distinguish our study from the previous study on simvastatin in MS [6], with its univariate analysis of mean values, although both use the same study design. In fact, this baseline-to-treatment trial design has been successfully used several times in the recent past to prove principles of treatment strategies in MS without the necessity for a long placebo period, the latter raising serious ethical concerns [17]–[19].

A further novelty in our study, besides the use of atorvastatin in MS, is the inclusion of patients with MRI activity despite IFN-β pre-treatment, who then received add-on medication. The scientific rationale for applying this combination therapy in this clinical trial originated from our observations of a clear immunomodulatory synergy of both drugs in culture assays. Indeed, applying multivariate analysis we observed a trend towards a significant reduction in CEL number and volume over time in the group with IFN-β comedication, but not in those patients undergoing atorvastatin monotherapy. However, sample sizes in both subgroups were too small and the differences in the statistical tests too minor to support the conclusion that combination therapy may be more efficacious than atorvastatin monotherapy. In addition, direct exploratory between-group comparisons (using two-factorial MANOVA) were unable to detect a more pronounced effect of the combination therapy on CEL number and volume. This discrepancy may be due to small and unequal sample size numbers in the two subgroups (24 vs. 12 patients), or to differences between the groups already at baseline, or may indeed reflect a non-superiority of the combination therapy. As our study was designed to assess longitudinal effects of atorvastatin, and not to compare groups of patients with and without IFN-β comedication, our data are not intended to draw a firm conclusion regarding treatment efficacy of a combination of atorvastatin with IFN-β. This conclusion is also undermined by other MRI parameters such as MTR and ADC, which only partially dichotomized the 2 subgroups in favour of the comedication group. On the other hand, the results of our MRI analyses argue against a possible detrimental effect of an atorvastatin/IFN-β combination therapy on disease course, as recently suggested [20]. This is supported by our clinical data: the decrease of the annualized relapse rate from the pre-treatment to the treatment period was comparable in both subgroups, and the number of relapses and proportion of patients suffering relapses while on atorvastatin treatment did not significantly differ between patients with and without IFN-β comedication. The same was also true of the change in EDSS and MSFC. Five patients dropped out of the study before completion of 6 months of atorvastatin treatment; two of them underwent combination therapy with IFN-β and experienced a relapse. It does not seem reasonable to hold the short-lasting combination treatment period responsible for disease exacerbation, all the more so as these patients also exhibited an active disease course prior to introduction of atorvastatin. Two further patients with IFN-β pre-treatment, who discontinued the study for reasons other than exacerbation after 5 months of atorvastatin treatment, did not experience relapses and displayed constant or even decreased CEL numbers/volume and T2 lesion load.

Moreover, according to our data, neither safety considerations nor side effects argue against a monotherapy with atorvastatin or a combination with IFN-β. Although certain adverse events, such as elevated CK, occurred more frequently in the IFN-β comedication group, only one patient from this group had to be withdrawn from the study due to sustained CK elevation; the second patient discontinuing atorvastatin for this same reason was in the atorvastatin monotherapy group. The proportion of patients experiencing an elevation of liver enzymes was comparable between both groups, arguing against any additional hepatotoxicity from combination therapy. Moreover, we noticed a reduced general tolerability, mostly evident in nausea and gastrointestinal side effects, irrespective of IFN-β pre-treatment, when immediately starting with 80 mg atorvastatin. These side effects required a temporary dose reduction and were therefore the principle reason for minor protocol violations. We therefore introduced a short run-in phase of 2 to 4 weeks, which should also be considered for further clinical trials with atorvastatin.

The underlying mechanisms through which statins may exert their beneficial influence on inflammatory activity in MS patients have not yet been fully elucidated. Previous data suggested a pronounced regulation of T lymphocytes, including a shift from T helper 1 to T helper 2 cells and direct interference in HMG-CoA-reductase-dependent T cell signalling pathways [4], [5], [21], [22]. Since we found neither a disturbed proliferative response nor an inhibition of pro-inflammatory cytokines in our patients, high-dose atorvastatin apparently does not exhibit overall peripheral immunosuppressive effects. An upregulation of IL-10, however, indicates an atorvastatin-mediated involvement of regulatory mechanisms *in vivo*.

In summary, we report that treatment with high-dose atorvastatin over 9 months was safe and well tolerated in the majority of our patients, regardless of IFN-β co-medication. Moreover, our data based on MRI surrogate measures for disease activity suggest possible beneficial effects of atorvastatin on lesion formation in patients with active disease. However, it remains to be investigated in future clinical trials whether the immunomodulatory effects observed here may indeed have an impact on the clinical disease course in RRMS. Thus, randomized, controlled trials with atorvastatin versus placebo given as an add-on to approved immunomodulators are warranted. Such a study design would meet both ethical concerns as well as scientific and methodological demands.

## Supporting Information

Checklist S1 CONSORT Checklist
(0.06 MB DOC)

Protocol S1 Trial Protocol
(0.22 MB PDF)
