# Supplementary material for: Oral High-Dose Atorvastatin Treatment in Relapsing-Remitting Multiple Sclerosis
Source: PLoS One. 2008 Apr 9;3(4):e1928. doi: 10.1371/journal.pone.0001928 (PMC2276246; doi:10.1371/journal.pone.0001928)
Supplement: Protocol S1 — Trial Protocol (0.22 MB PDF) [file pone.0001928.s002.pdf]

Antrag für Ethikkommission

„Investigator-driven“ Offene „Baseline-to-Treatment“ „Cross-Over“ MRT-kontrollierte Monozentrische Pilot Phase II Studie bei Patienten mit schubförmig-remittierender Multipler Sklerose (RRMS) zur Evaluation der Wirkungen, die in monatlichen Kernspin (MRT)-Untersuchungen zu sehen sind, der Schubrate und der Progression sowie des Wirkmechanismus im Patienten von dem Lipidsenker Atorvastatin, der täglich über neun Monate oral gegeben wird.

Autor: Prof. Dr. F. Zipp **Datum: 16.4.2003**

Leiter der klinischen Prüfung: Prof. Dr. F. Zipp, Leiterin des Instituts für Neuroimmunologie, Experimentelle und Klinische Neuroimmunologie (s. CV)

Beteiligte Ärzte/Wissenschaftler/Studienschwester des Instituts für Neuroimmunologie:

Frau J. Bellmann-Strobl, Wissenschaftliche Assistentin, Ärztin
Frau U. Wegener, stellvertretende ärztliche Leiterin
Herr J. Würfel, Wissenschaftlicher Assistent, Arzt (MRT-Studien-Ausbildung bei Prof. H. McFarland, am NIH, Bethesda, Maryland, USA)
Frau S. Waiczies, Post-Doktorandin, Pharmakologin (Promotionsarbeit eingereicht)
Frau B. Kreuzer (Studienschwester)

Stellungnahme zu finanziellen Voraussetzungen und Durchführbarkeit:

Diese Studie wird als „Research Grant“ von Pfizer mit 80.000 Euro unterstützt, die Medikation wird bereitgestellt, die Hauptkosten für Personal, Labor und MRT werden vom Institut für Neuroimmunologie übernommen. Alle für die Durchführbarkeit der Studie notwendigen Voraussetzungen einschließlich Kapazitäten für Patientenversorgung und MRT-Untersuchungen sind vollumfänglich im Institut für Neuroimmunologie vorhanden. Andere Studien mit gleichem Indikationsgebiet werden zeitgleich vom Institut für Neuroimmunologie nicht durchgeführt.

Es wurde kein Antrag in gleicher Sache bei einer anderen Ethikkommission gestellt.

Übersicht

Studienphase	Phase II
Produkt, Dosierung und Verabreichungsart	Atorvastatintabletten mit 80 mg oral/die
Indikation	“Relapsing-remitting” Multiple Sklerose (RRMS)
Ziele der Studie	Wirkungen auf MRT-Parameter, Klinik und immunologische Parameter von oral verabreichtem Atorvastatin, Vergleich einer „Baseline“-Periode zur Behandlungsphase
Patientenpopulation	Männliche und weibliche Patienten, 18 – 55 Jahre alt; EDSS 0 – 6; bei der Selektion im MRT mindestens eine Kontrastmittel-anreichernde Läsion in der T1-Wichtung und mindestens ein deutlicher, dokumentierter Schub innerhalb 12 Monaten (bei einer IFN-beta Behandlung von weniger als 12 Monaten muß der Schub 3 Monate nach Beginn der IFN-beta Therapie erfolgt sein) vor der Rekrutierung
Studiendesign	Offen, Pilotphase, „Baseline-to-treatment”, “Cross-Over”, monozentrisch, Phase II
Behandlungsdauer	9 Monate („Baseline“ 3 Monate)
Methodologie	Primär MRT: Anzahl an Gd-aufnehmenden Läsionen
Die Gesamtzahl an Patienten, die statistische Begründung	50; die Größe der Stichprobe ist mit einer statistischen Fallzahlplanung begründet, um die Wirksamkeit des primären MRT-Parameters bewerten zu können
Unerwünschte unerwartete Ereignisse („Adverse Events“=AE)	Von den Patienten spontan berichtet; Labor- und ärztliche Untersuchung; diese werden der Ethikkommission und dem KKS an der Charité gemeldet. Eine Interims-Analyse erfolgt nach 3 Monaten Behandlung.
Plan der Datenanalyse	Diese Studie analysiert primär die Wirksamkeit von Atorvastatin, weitere sekundäre Endpunkte werden explorativ untersucht.
Geplanter Beginn und Ende der Rekrutierung	Juni 2003 – Mai 2004

1. Ziel des Vorhabens

1.1. Hypothese:

Atorvastatin reduziert die MRT-Aktivität, die Schubrate und Progression bei Patienten mit schubförmig-remittierender Multipler Sklerose und wirkt über die Regulation des T-Zell-Zyklus sowie über Entzündungshemmung.

1.2. Einleitung

Multiple Sklerose (MS) ist die häufigste, nicht traumatische Ursache von neurologischer Behinderung bei jüngeren Menschen. Die meisten Patienten entwickeln eine schubförmig-remittierende Form der MS (RRMS), bei der schubförmig auftretende neurologische Symptome von kompletten oder inkompletten Remissionen gefolgt werden. Etwa 60-70% der Patienten mit RRMS entwickeln dann später einen sekundär-chronisch progredienten Verlauf (SPMS), eine Erkrankung, die durch eine zumeist schleichende Progression einer neurologischen Behinderung mit oder ohne überlagerte Schübe charakterisiert ist. Anerkannte Medikamente für die Behandlung von RRMS wie z. B. Beta-Interferone oder Glatiramerazetat reduzieren die Schubrate um etwa 30%, können aber nur parenteral gegeben werden und zeigen einige unerwünschte Nebenwirkungen (Grippe-ähnliche Symptome, lokale Unverträglichkeit, u.s.w.). Ein Medikament, welches als Monotherapie zum Abbremsen der Progression der Erkrankung bei Patienten mit RRMS oral gegeben werden könnte, wird dringend gebraucht und wäre ein wichtiger Schritt vorwärts in der MS-Therapie.

Eine ungewollte Aktivierung und Vermehrung von T-Lymphozyten kann zu ausgedehnter Inflammation und Gewebeschädigung führen, den entscheidenden pathophysiologischen Elementen mehrerer Autoimmunkrankheiten einschließlich der MS. Daher ist eine therapeutische Strategie die spezifische Eindämmung von T-Zell-Aktivität, -Vermehrung und -Effekten, ohne daß es zu der unspezifischen Zell-Schädigung kommt, die die in der MS bedingt wirksamen nicht-selektiven Immunsuppressiva (Zytostatika mit erheblichen Nebenwirkungen) hervorrufen. Zu den Substanzen, die wesentlich schonender und spezifischer auf die T-Zellen Einfluß nehmen als Zytostatika, gehören bereits sowohl die Beta-Interferone als auch das Glatiramerazetat. Insbesondere die Beta-Interferone führen neben den obengenannten klinischen Auswirkungen zu einer schnellen Reduktion der Kontrastmittel (KM- oder Gadolinium[Gd]-Diethylen-triamin-pentaacetat[DTPA])-aufnehmenden Läsionen im MRT, die zwar nicht genau mit der Klinik korrelieren, jedoch ebenfalls ein Maß für die Aktivität der Erkrankung sind.
Zahlreiche therapeutische Interventionsstudien belegen einen günstigen prophylaktischen Effekt von Statinen auf das Herzinfarkt- und Schlaganfallrisiko (Maron et al., 2000). Diese Risikoreduktion wurde bislang durch ihren Lipid-senkenden Einfluß über die Hemmung der β-HMG-CoA-Reduktase erklärt, eines für die Cholesterolsynthese essentiellen Enzyms. Neuere klinische und experimentelle Studien legen allerdings nahe, daß Statine auch antiinflammatorische Eigenschaften haben. Nach zwei unabhängigen Studien senken Pravastatin und Lovastatin den CRP-Spiegel im Serum von Patienten mit koronarer Herzerkrankung um ca. 15% (Albert et al., 2001; Ridker et al. 2001). Für die Prävention der Transplantatabstoßung bei Herzverpflanzungen konnte gezeigt werden, daß Pravastatin die Abstoßungsrate reduziert (Kobashigawa et al., 1995). Nach *in vitro* Versuchen von Kwak et al. (Kwak et al., 2000) hemmen Statine, hierunter vor allem Atorvastatin, die für die professionelle Antigenerkennung wichtige, induzierbare Expression von MHC Klasse II Molekülen auf Antigen-präsentierenden Zellen (Makrophagen, B-Zellen). Dies könnte im Fall von Autoimmunkrankheiten bedeuten, daß der für die adäquate Stimulation autoreaktiver T-Zellen nötige Antigenerkennungsprozeß nicht stattfindet. Entsprechend konnten Stanislaus et

al. (Stanislaus et al., 1999 u. 2001) in einem rein inflammatorischen, monophasischen Ratten-Modell der EAE, induziert in Lewis-Ratten durch Immunisierung mit Myelin-basischem Protein (MBP), die klinische Wirksamkeit von Lovastatin demonstrieren. Weitere *in vivo* Studien in Mäusen mit chronisch-schubförmiger EAE, die immunologisch und neuropathologisch dem humanen Pendant eher ähnelt, zeigten einen signifikanten protektiven Effekt von Atorvastatin (Youssef et al., 2002). In eigenen *in vivo* EAE-Untersuchungen gelang der Nachweis, daß Atorvastatin auch bei therapeutischer Applikation, also verabreicht nach Krankheitsmanifestation, ein Krankheitsrezidiv („Schub“) verhindern kann (Aktas et al., 2003). Nicht nur in murinen, sondern vor allem in humanen Antigen-spezifischen T-Zellen konnten wir einen MHC-unabhängigen antiproliferativen Effekt von Atorvastatin zeigen (Aktas et al., 2003). Dieses Statin greift in den Zellzyklus ein und hemmt die Expression von Zyklin-abhängiger Kinase 4 (CDK4), indem es $p27^{kip1}$ heraufreguliert, einen für Anergie wichtigen Faktor. Dieser Effekt ist HMG-CoA-Reduktase-abhängig, was wir durch die Reversibilität mit L-Mevalonat nachweisen konnten, und ist möglicherweise auf die Fähigkeit der posttranslationalen Modifikation (Isoprenylierung) kleiner GTP-bindender Moleküle durch Statine zurückzuführen. Die *in vivo* und *in vitro* Ergebnisse machen den Einsatz von Statinen in der Therapie der schubförmigen MS plausibel, zumal durch die orale Formulierung ein weiterer Vorteil gegenüber aktuellen Behandlungsformen gegeben ist und die Verträglichkeit der meisten Statin-Derivate durch den breiten langjährigen Einsatz als Lipidsenker bei der Behandlung der koronaren Herzkrankheit gut gesichert ist. Wie einem bereits im Internet zu lesenden Beitrag auf der diesjährigen Tagung der amerikanischen neurologischen Gesellschaft zu entnehmen ist, wurden bereits 45 MS-Patienten 6 Monate lang mit Simvastatin, einem anderen Statin, behandelt. Die Studie zeigte bei einer 3-monatigen Periode vor Therapiebeginn und 6-monatiger Behandlungsphase eine Reduktion der Gd-aufnehmenden Läsionen im MRT (Durchschnitt/SD 2.35/1.94 vor und 1.31/1.30 nach Behandlung; p=0.0001) (Vollmer et al., 2003).

Referenzen:

- Aktas O, Waiczies S, Smorodchenko A, Dörr J, Seeger B, Prozorovski T et al. Treatment of relapsing paralysis in experimental encephalomyelitis by targeting Th1 cells through atorvastatin. J Exp Med 2003 (in press).
- Albert MA, Danielson E, Rifai N, Ridker PM. Effect of statin therapy on C-reactive protein levels: the pravastatin inflammation/CRP evaluation (PRINCE): a randomized trial and cohort study. JAMA 2001; 286:64-70.
- Kobashigawa JA, Katznelson S, Laks H, Johnson JA, Yeatman L, Wang XM et al. Effect of pravastatin on outcomes after cardiac transplantation. N Engl J Med 1995; 333:621-627.
- Kwak B, Mulhaupt F, Myit S, Mach F. Statins as a newly recognized type of immunomodulator. Nat Med 2000; 6:1399-1402.
- Maron DJ, Fazio S, Linton MF. Current perspectives on statins. Circulation 2000; 101:207-213.
- Ridker PM, Rifai N, Clearfield M, Downs JR, Weis SE, Miles JS et al. Measurement of C-reactive protein for the targeting of statin therapy in the primary prevention of acute coronary events. N Engl J Med 2001; 344:1959-1965.
- Stanislaus R, Pahana K, Singhb AK, Singh I. Amelioration of experimental allergic encephalomyelitis in Lewis rats by lovastatin. Neurosci Lett 1999; 269:71-74.
- Stanislaus R, Singh AK, Singh I. Lovastatin treatment decreases mononuclear cell infiltration into the CNS of Lewis rats with experimental allergic encephalomyelitis. J Neurosci Res 2001; 66:155-162.

• Vollmer T, Durkalski V, Tyor W, Corboy J, Preiningerova J, Markovic-Plese S et al. An open-label, single arm study of simvastatin as a therapy for multiple sclerosis (MS). AAN 2003
• Youssef S, Stüve O, Patarroyo JC, Ruiz PJ, Radosevich JL, Hur EM et al. The HMG-CoA reductase inhibitor, atorvastatin, promotes a Th2 bias and reverses paralysis in central nervous system autoimmune disease. Nature 2002; 420:78-84.

1.3 Begründung der Studie

In dieser Studie werden Wirkungen, primär erfaßt durch MRT und sekundär durch Schubrate, Progression und immunologische Parameter, von Atorvastatin auf die MS-Krankheitsaktivität in Patienten mit RRMS untersucht. Die Abnahme von aktiven MS-Läsionen (also Kontrastmittel [Gd]-aufnehmenden), wie sie in fortlaufenden MRTs zu sehen sind, wird zur Zeit als der sensitivste vorhandene Marker betrachtet, um die entzündungshemmende Aktivität einer potentiellen MS-Behandlung darzustellen. Aus diesem Grund ist ein MRT Endpunkt optimal für eine Phase II Studie.

Anerkannte Arzneimittel wie Beta-Interferone (IFN-beta) oder Glatiramerazetat für die Behandlung von RRMS reduzieren die Schubfrequenz um etwa 30%, können aber nur parenteral verabreicht werden und zeigen einige unerwünschte Nebenwirkungen. Eine orale Applikation von Atorvastatin wäre viel günstiger für Patienten, die eine dauerhafte Behandlung erhalten. Außerdem stellt diese Substanz durch den direkten Eingriff in die zur T-Zell-Vermehrung führende Signalkaskade ein neues Behandlungsprinzip für Autoimmunkrankheiten dar.

Das bereits als Lipidsenker zugelassene Medikament Atorvastatin hat als Nebenwirkungen gelegentlich Obstipation, Dyspepsie, Bauchschmerzen, Übelkeit, Myalgien, Asthenie, Durchfall. Ebenfalls gelegentlich kommt es zu einem CK-Anstieg, selten verbunden mit Muskelschmerzen, Empfindlichkeit u. Schwäche der Muskulatur.

Diese Studie ist konzipiert, um die Wirkungen der Gabe von 80 mg Atorvastatin täglich über 9 Monate bei RRMS Patienten mit und ohne Interferon-beta Therapie zu prüfen. Die Sicherheit und Verträglichkeit von Atorvastatin wird regelmäßig durch klinische Auswertungen und Routinelaborparameter bewertet. Die Wirksamkeit der Substanz in der MS wird durch monatliches MRT, die monatliche neurologische Untersuchung (MSFC = Multiple Sklerosis Functional Composite Score), EDSS (Expanded Disability Status Scale) in größeren Abständen und Erfassung von Schüben sowie immunologische Untersuchungen zur T-Zell-Regulation geprüft.

Das primäre Zielkriterium ist die kumulative Zahl der neu auftretenden aktiven, also Gd-aufnehmenden, Läsionen im MRT während der Behandlung im Vergleich zur Periode vor Behandlung.

Darüber hinaus werden mehrere andere MRT-, klinische und immunologische Variablen untersucht (s. 3.).

2. Sicherheitsdaten und -maßnahmen

Das bereits als Lipidsenker zugelassene Medikament Atorvastatin hat als Nebenwirkungen gelegentlich Obstipation, Dyspepsie, Bauchschmerzen, Übelkeit, Myalgien, Asthenie, Durchfall. Ebenfalls gelegentlich kommt es zu einem CK-Anstieg, selten verbunden mit Muskelschmerzen, Empfindlichkeit u. Schwäche der Muskulatur. Ohne direkten Zusammenhang mit Atorvastatin: selten Muskelkrämpfe, Myositis, Myopathie, Rhabdomyolyse, Parästhesien, periphere Neuropathien, Pankreatitis, Hepatitis, cholestatischer Ikterus, Anorexie, Erbrechen, Alopezie, Pruritus, Hautausschlag, Impotenz, Hyperglykämie od. Hypoglykämie, Schmerzen in der Brust, Schwindel, Thrombozytopenie, allerg. Reakt. u. angioneurotisches Ödem. Außerdem werden Gelenkschmerzen und bullöses Exanthem angegeben.
Das Risiko, eine Myopathie zu entwickeln, war bei Kombination anderer HMG-CoA-Reduktase-Hemmer mit Cyclosporin, Fibraten, Erythromycin, Antimykotika vom Azol-Typ oder Nikotinsäurederivaten (Niacin) erhöht, wobei selten eine Rhabdomyolyse mit Nierenversagen als Folge einer Myoglobinurie aufgetreten ist. Erythromycin, 4× tgl. 500 mg bei gesunden Personen erhöht den Atorvastatin-Plasmaspiegel. Bei Gabe von oralen Kontrazeptiva kommt es unter Atorvastatingabe zu einem Anstieg von Norethisteron u. Ethinyl-Estradiol. Colestipol senkt den Atorvastatin-Plasmaspiegel um ca. 25%. Antacida (Magnesium- und Aluminiumhydroxid) senken den Atorvastatin-Plasmaspiegel um ca. 35%.

Kontraindiziert ist die Gabe von Atorvastatin bei Frauen im gebärfähigen Alter, die keine geeigneten Verhütungsmethoden anwenden und bei Jugendlichen bis 18 Jahren. Patienten mit aktiver Lebererkrankung und solche mit Myopathie dürfen das Medikament nicht einnehmen. Während der Stillzeit ist es ebenfalls kontraindiziert. Eine Anwendungsbeschränkung besteht bei starkem Alkoholkonsum sowie gleichzeitiger Anwendung von Hemmstoffen des Cytochrom P 450 3A (z. B. Cyclosporin, Makrolidantibiotika und Antimykotika vom Azol-Typ).

Den Patienten wird mitgeteilt, dass sie Grapefruchtsaft nicht in großen Mengen trinken sollen, da dieser die Plasmaspiegel von Atorvastatin erhöhen kann.

Um sich ausreichend mit den möglichen, noch nicht identifizierten Risiken für die Patienten zu befassen, werden folgende Sicherheitsmaßnahmen deshalb ergriffen:

- Vor der Behandlung: Patienten, die eine dauerhafte Behandlung benötigen mit Medikamenten, die Cytochrom P 450 A3 hemmen und die durch alternative Medikamenten nicht zu ersetzen sind, werden nicht in die Studie einbezogen.
- Während der Behandlung: Patienten, die eine neue, begleitende Behandlung mit Medikamenten benötigen, die Cytochrom P 450 A3 hemmen, scheiden aus der Studie aus, es sei denn, diese Medikamente können durch ein entsprechendes Arzneimittel ersetzt werden, das nicht Cytochrom P 450 A3 hemmt.

Für die an der Studie teilnehmenden Patienten wird eine Patientenversicherung gemäß §40 (1) Nr. 8 und (3) AMG sowie §20 (1) Nr. 9 und (3) MPG abgeschlossen. Derzeitig werden durch die Abt. Material- und Versorgungswirtschaft, Grundstück- und Versicherungswesen (Frau I. Brune) Kostenvoranschläge eingeholt.

3. Prüfplan bzw. Studiendesign

3.1. Beschreibung des Studiendesigns bzw. -plans

- „Investigator-driven“ Offene „Baseline-to-Treatment“, „Cross-Over“, MRT-kontrollierte Monozentrische Pilot Phase II Studie bei Patienten mit RRMS.

- Teilnahme von 50 Patienten, die ab Bewilligung des Ethikvotums rekrutiert werden. Es dürfen nur die Patienten für die Studie rekrutiert werden, die eine unterschriebene Einwilligung zur Verfügung gestellt haben.

- Patienten werden nach IFN-beta-Komedikation in zwei gleichgroße Strata stratifiziert.

- Jede individuelle Studie soll 12 Monate umfassen, davon 3 Monate ohne Therapie und 9 Monate unter Therapie.

- Jeder Patient besucht das Studienzentrum für 13 regelmäßige Termine: 4 Termine vor Therapiebeginn, 9 Termine unter Therapie.

- Nach 3 Monaten Therapie erfolgt eine Interimsanalyse.

- Patienten, die frühzeitig aus der Studie ausscheiden, werden bis Ende der Studie bei den festgelegten Terminen beobachtet, es sei denn die Einwilligung wird zurückgezogen oder der/die Patient/in kann nicht mehr verfolgt werden.

- Diese Studie wird gemäß dem Protokoll geführt, in Übereinstimmung sowohl mit den ethischen Prinzipien der Declaration of Helsinki und den ICH-GCP Rechtlinien vom 17.1.1997 als auch mit den Vorschriften der hiesigen Ethikkommission.

3.2. Diskussion über Studiendesign

Dieses „Baseline-to-Treatment“ „Cross-Over“ Design ist bei dem sehr heterogenen klinischen Verlauf der MS für eine Phase II Pilotstudie zur Wirksamkeitsprüfung geeignet, eine interne Kontrollgruppe durch die „Baseline“ zu haben, um zunächst die ethisch schwierigere Plazebo-kontrollierte Studie zu vermeiden. Durch geblindete Auswertung von objektivierbaren MRT-Parametern, die über einen Zeitraum von 9 Monaten sensitiver sind als die klinische Untersuchung, ist in der Pilotphase bereits eine zuverlässige Aussage über die Wirksamkeit des Medikaments möglich. (S. dazu auch die Multiple Sklerose-Studien-Internet-Seite der National Institutes of Health, NIH, NINDS, Bethesda, Maryland, USA.)
Die Rekrutierung von sowohl unbehandelten als auch mit IFN-beta vorbehandelten Patienten, bei denen IFN-beta nicht ausreichend wirksam ist, erlaubt zusätzlich die Aussage, ob Atorvastatin möglicherweise sogar wirksamer in der Kombination mit dem etablierten immunmodulatorischen IFN-beta ist. Beide subkutan verabreichten IFN-beta-Präparationen (Rebif, Betaferon) werden als Vorbehandlung gestattet, da sie etwa gleich in der Dosierung und Wirksamkeit sind.

In die Studie sollen insgesamt 50 Patienten (25 mit- und 25 ohne IFN-beta-Vorbehandlung) einbezogen werden.

3.3. Selektion der Studienpopulation

Selektiert als Population für diese klinische Studie werden ambulante RRMS Patienten mit einer aktiven Erkrankung, die im MRT durch mindestens eine Gd-DTPA-aufnehmende Läsion in der T1-gewichteten Sequenz sowie durch wenigstens einen deutlichen, dokumentierten Schub innerhalb von 12 Monaten (bei einer IFN-beta Behandlung von weniger als 12 Monaten muß der Schub 3 Monate nach Beginn der IFN-beta Therapie erfolgt sein) vor der Rekrutierung bestätigt ist. Für den Fall einer IFN-beta Behandlung, die bereits mindestens 6 Monate erfolgt sein muß, ist dies gleichbedeutend mit einer nicht optimalen Wirksamkeit des IFN-beta.
Hinsichtlich nachfolgender MRT-Aktivität hat sich diese Patientenpopulation als die am meisten sensitive herausgestellt. Ein/e einzige/r Patient/in darf nur einmal an einer solchen Studie teilnehmen.

3.4. Einschlußkriterien

Jeder Patient muß folgende Einschlußkriterien erfüllen:

1. Lebensalter zwischen 18 und 55 bei der Rekrutierung
2. die Diagnose MS entsprechend den McDonald Kriterien
3. MS mit schubförmig-remittierenden Verlauf
4. EDSS 0 – 6
5. bei der Rekrutierung eine aktive Erkrankung, die folgendermaßen bestätigt werden kann: im MRT mindestens eine Gd-aufnehmende Läsion in T1-gewichteter Sequenz und mindestens ein deutlicher, dokumentierter Schub innerhalb von 12 Monaten vor der Rekrutierung (bei einer IFN-beta Behandlung von weniger als 12 Monaten muß der Schub 3 Monate nach Beginn der IFN-beta Therapie erfolgt sein)
6. eine Therapie mit IFN-beta muß mindestens 6 Monate erfolgt sein
7. Frauen im gebärfähigen Alter müssen sich bereit erklären, ausreichende Schwangerschaftsverhütung zu praktizieren; alle Frauen müssen einen negativen Schwangerschaftstest nachweisen
8. eine voll-informierte, schriftliche Einwilligung muß vorgelegt werden

3.5. Ausschlußkriterien

Patienten mit folgenden Kriterien dürfen nicht eingeschlossen werden:

1. eine primär chronisch-progrediente MS-Form
2. jegliche andere Erkrankung als MS, die die Zeichen und Symptome der/s Patientin/en besser erklären könnte
3. jegliche Konditionen, die das MRT oder andere Auswertungen stören oder verhindern könnten
4. klinisch relevante Magen-Darm-Erkrankungen wie z.B. Colitis ulcerosa, Morbus Crohn; Krankengeschichte von Ulcus pepticum
5. klinisch relevante Lungen-, infektiöse, Herz- oder andere ZNS-Erkrankungen
6. klinisch relevante Leber-, Nieren-, oder Knochenmarksfunktionsstörungen, wie sie durch die folgenden Laborwerte definiert sind:
   - Knochenmarksdysfunktion: Hb < 8,5 g/dl
     WBC < 2,5 x 10^9/L
     Blutplättchenzahl < 125 x 10^9/L
   - Nierendysfunktion: Kreatinin > 180µmol/L

- Leberdysfunktion: ASAT (SGOT) > 3 x höher als Normalbereich
  Bilirubin ≥ 2 x höher als Normalbereich

7. bekannte Allergie gegen Gd-DTPA
8. bekannte Überempfindlichkeit gegen Bestandteile des Arzneimittels
9. Teilnahme an jeglicher klinischen Studie innerhalb der letzten 6 Monate oder während der gesamten Studiendauer
10. Vorbehandlung mit folgenden Substanzen vor der Rekrutierung in die Studie innerhalb folgender Zeiträume:
    - vollständige Lymphbestrahlung; Antilymphozyten-Antikörper-Behandlung (z.B. Anti-CD4, Campath-1H)
    - 6 Monate vor dem Studienbeginn: Mitoxantron, Cyclophosphamid, Cyclosporin A, humane Antikörper, alle anderen immunmodulierenden oder immunsupprimierenden Substanzen einschließlich anderer rekombinanter Zytokine, oder anderer potentieller oder experimenteller MS-Behandlung, (ausschließlich Interferon-beta in dem hier untersuchten Behandlungsarm mit Interferon-beta als Komedikation)
    - 3 Monate vor dem Studienbeginn: Glatirameracetat, Azathioprin, IVIg
11. Schwangerschaft oder Laktation
12. Alkohol- oder Drogenmißbrauch
13. Hemmstoffe des Cytochrom P 450 3A (z. B. Cyclosporin, Makrolidantibiotika und Antimykotika vom Azol-Typ).
14. Medizinische, psychiatrische oder andere Konditionen, die die folgende Fähigkeiten der/s Patientin/en einschränken: die Patienteninformation zu verstehen, informierte Einwilligung zu geben, sich an die Regeln des Protokolls zu halten oder die Studie zu vollenden.

3.6. Der Abbruch der Behandlung

Alle Patienten haben zu jeder Zeit das Recht, die weitere Teilnahme an der Studie abzulehnen, ohne dafür eine Begründung zu geben. Die Teilnahme einer/s Patientin/en ist auf deren/dessen Wunsch unverzüglich abzubrechen. Der Untersucher sollte sich bemühen, den Grund herauszufinden, und diesen in der Studienakte einzutragen.

Sollte ein/e Patient/in seine/ihre Teilnahme an der Studie beenden wollen, aber schon mindestens eine Dosis des Studienmedikaments genommen haben, wird ihm oder ihr empfohlen, die vereinbarten Termine für die verbleibende Studiendauer wahrzunehmen, es sei denn, er oder sie entzieht die Einwilligung oder Nachuntersuchungen sind nicht mehr möglich.

Ein/e Patient/in kann nach Ermessen des Leiters der klinischen Prüfung aus der Studie ausscheiden. Der Grund dafür sollte in der Studienakte umfassend dokumentiert werden. Sollte der/die Patient/in im Laufe der Studie einen Zustand entwickeln, der ihre/seine Teilnahme an der Studie nach den Ausschlusskriterien verhindert hätte, muss er oder sie die Studie unverzüglich abbrechen. Die Begründungen dafür sollten in der Studienakte umfassend dokumentiert werden.

Der Untersucher muss einen Studienabbruch in Erwägung ziehen, sollte ein/e Patient/in Medikamente für eine begleitende Behandlung benötigen, die Hemmstoffe des Cytochrom P 450 3A (z. B. Cyclosporin, Makrolidantibiotika und Antimykotika vom Azol-Typ).

Der Untersucher sollte einen Abbruch der Behandlung einer/s Patientin/en in Erwägung ziehen im Falle eines unerwarteten unerwünschten Ereignisses („Adverse Events“=AE) oder eines schwerwiegenden solchen Ereignisses („Serious Adverse Event“=SAE), oder im Falle klinisch relevanter Laborabweichungen.

Beim Vorkommen isolierter Lymphozyten-, CK- oder Transaminasen-Pathologie ohne klinische Zeichen oder Symptome soll die Studienmedikation unterbrochen werden, bis Normalisierung erreicht wird. Die Behandlung darf dann wieder aufgenommen werden, allerdings nur unter strikter Beobachtung. Sollte es zu einer erneuten Verschlechterung dieser Werte kommen, dann muß die Behandlung unterbrochen werden und ein Entzug aus der Studie in Erwägung gezogen werden.

Außerdem muß die Behandlung einer/s Patientin/en mit dem Studienmedikament endgültig abgebrochen werden in folgenden Situationen:

- Nicht autorisiertes Nutzen des Studienmedikaments

- Schwangerschaft

- keine Nachuntersuchungen mehr möglich
  Anmerkung: Wenn bei einer/em Patientin/en mehr als zwei aufeinander folgende Termine ausfallen, ohne daß es dafür einen Grund gibt, mit dem der Leiter der klinischen Prüfung auch einverstanden ist, dann gilt diese/r Patient/in als nicht mehr für Nachuntersuchungen vorhanden.

- Teilnahme an anderen klinischen Studien im Laufe dieser Studie

- Der Beginn anderer pharmakologischen MS-Behandlungen einschließlich systemischer Steroidtherapie, falls systemische Steroidtherapie nicht als Behandlung für einen akuten Schub angewendet wird.

3.7. „Adverse Events“ (unerwartete unerwünschte Ereignisse) (AE)

Alle unerwünschten Ereignisse werden auf dem AE-Bogen in der Studienakte bei allen Besuchen erfasst. Eine Veränderung in der Intensität eines AE macht eine zusätzliche AE-Seite nötig. Einzelheiten hierzu werden in 5.3. beschrieben.

Wenn Patienten aus irgendeinem Grund ins Krankenhaus eingewiesen werden, soll die Verabreichung der Studienmedikation fortgesetzt werden, es sei denn der/die Untersucher/in empfiehlt das Absetzen. Jeder Krankenhausaufenthalt stellt einen „Serious Adverse Event“ (schwerwiegendes unerwartetes unerwünschtes Ereignis) (SAE) dar.

3.8. Behandlung

Beim 4., 8., und 11. Termin werden die Patienten mit Tabletten für 3 Monate versorgt. Das Etikettieren übernimmt die Charité-Apotheke. Patienten werden angewiesen, die leeren Packungen und unbenutzten Medikamente dem Studienzentrum zurückzugeben. Der klinische Untersucher wird die zurückgebrachten Medikamente überprüfen. Jegliche Diskrepanzen

werden dokumentiert. Die Behandlung beginnt am Tag 1 nach der Monat 0-Untersuchung, sobald ein negatives Ergebnis des Schwangerschaftstests (beta-HCG Blutprobe) vorliegt.

Alle begleitenden Medikamente sowie jede Veränderung derer oder Hinzutreten neuer Medikamente während der Studienzeit müssen an jedem Termin abgefragt und in der Studienakte eingetragen werden. Ganz am Anfang müssen auch begleitende Medikamente dokumentiert werden, die in den letzten 2 Wochen vor der Annahme der Studienmedikation genommen wurden. Die Patienten werden in der Patienteninformation darauf hingewiesen, dass sie neue Medikamente nur nach Rücksprache nehmen sollen. Medikamente, die Hemmstoffe des Cytochrom P 450 3A (z. B. Cyclosporin, Makrolidantibiotika und Antimykotika vom Azol-Typ) sind, dürfen während der Studie nicht eingenommen werden.

3.9. Vitalfunktionen/Ärztliche Untersuchung

Vor und am Ende der Behandlung werden Vitalfunktionen wie Herzfrequenz, systolischer und diastolischer Blutdruck (sitzend nach 3 Minuten) gemessen. Bei klinisch relevanten Abweichungen in den Meßwerten, sollen diese vor der Dokumentation wiederholt werden. Ebenso werden das allgemeine Aussehen, Haut, Augen, Ohren, Nase, Hals (einschließlich der Schilddrüse), Lungen, Herz, Brüste, Bauch (Magen-Darm-Trakt, Stuhlgewohnheiten; und Leber, Nieren, Milz, u.a.), Lymphknoten, und Skelettmuskelsystem (inkl. die Extremitäten und Wirbelsäule) untersucht. Wenn es wegen der medizinischen Geschichte des/der Patienten/in angebracht ist, werden das Urogenitalsystem, die gynäkologische Organe und das Rektum von Spezialisten untersucht.

3.10. Expanded Disability Status Scale (EDSS)

Der EDSS wird zum einen bei der Rekrutierung und zum anderen zu den Zeitpunkten 0, 1, 3, 6, 9 Monate durchgeführt. Die EDSS-Bewertung basiert auf einer standardisierten neurologischen Untersuchung der wesentlichen Funktionssysteme (optische, Hirnstamm-/Hirnnerven-, pyramidale, zerebelläre, sensible, vegetative und übergeordnete zerebrale Funktionen).

3.11. Multiple Sclerosis Functional Composite (MSFC)

Der MSFC wird bei jeder Vorstellung insgesamt 13 Mal (Zeitpunkte –3 bis 9) durchgeführt. Er besteht aus drei Teilen („9-Hole Peg Test“ Steckbrett-Test, „Timed 25-Feet Walk Test“ gestoppte Gehstrecke über 8 m, „Paced Auditory Serial Addition Test“ akustischer serieller Rechentest) und stellt ein standardisiertes quantitatives Instrument dar, objektiviert die Funktion der oberen und unteren Extremitäten sowie die kognitive Funktion zu messen.

3.12. Schübe

Ein Schub ist das Auftreten einer neuen neurologischen Abnormität oder das Wiederauftreten von neurologischen Symptomen, das einer Periode von mindestens 30 Tagen eines stabilen oder sich bessernden neurologischen Zustands folgt. Die neu aufgetretene Symptomatik muß mindestens 24 Stunden dauern und unabhängig von Fieber, bekannten Infektionen oder Absetzen einer systemischen Steroidbehandlung vorkommen.

Die Patienten werden angewiesen, das Studienzentrum innerhalb 24 Stunden zu kontaktieren, sollten irgendwelche Symptome, die auf einen Schub hinweisen, vorkommen. Das Zentrum soll die/den Patient/in bald möglichst innerhalb der darauf folgenden sieben Tagen evaluieren. Betroffene neurologische Funktionen werden erfaßt. Nur Schübe, die von einem/r Untersucher/in bestätigt wurden, werden als gültig betrachtet (einschließlich die, die von einem/r anderen Arzt/Ärztin früher bestätigt wurden, wenn diese in den Patientenakten sachgerecht dokumentiert sind). Alle notwendigen Untersuchungen werden in der Studienakte dokumentiert. „Follow-up“ Besuche werden nach Ermessen des/r Untersuchers/in festgelegt, um die Entwicklung des Schubs zu überwachen.

3.13. Sicherheits- und Wirksamkeitsvariablen

Alle für diese Studie nötige Verfahren, Proben und Untersuchungen sind in der folgenden Tabelle aufgelistet.

Ereignis	Monat –3	Monat -2	Monat -1	Tag der 1. Tabl. Vor	Nach	Monat 1	Monat 2	Monat 3	Monat 4	Monat 5	Monat 6	Monat 7	Monat 8	Monat 9
Einwilligungserklärung	X													
Demographische Daten [1)]	X													
Menstruationsanamnese	X													
Schwangerschaftsverhütung	X	X	X	X		X	X	X	X	X	X	X	X	X
Schwangerschaftstest	X			X[9)]										
Diagnose MS	X													
Anamnese MS	X													
Anamnese von Schüben	X													
Medizinische und chirurgische Anamnese	X													
Medikamentenanamnese	X													
Ärztliche Untersuchung	X			X		X		X			X			X
Vitalwerte[2)]	X			X		X		X			X			X
Neurologische Untersuchung (EDSS)	X			X		X		X			X			X
MSFC	X	X	X	X		X	X	X	X	X	X	X	X	X
EKG	X													X
Erweitertes Labor[3)]	X			X										X
Großes Routinelabor[4)]						X					X			
Kleines Routinelabor[5)]								X						
Immunologie[6)]	X		X	X		X		X			X			X
MRT Routine[7)]	X	X	X	X		X	X	X	X	X	X	X	X	X
MRT Erweitert[8)]	X			X				X			X			X
Einschluß-/ Ausschlusskriterien	X			X										
Abfrage von begleitenden Medikamenten	X	X	X	X		X	X	X	X	X	X	X	X	X

Ereignis	Monat –3	Monat -2	Monat -1	Tag der 1. Tabl. Vor	Nach	Monat 1	Monat 2	Monat 3	Monat 4	Monat 5	Monat 6	Monat 7	Monat 8	Monat 9
Nebenwirkungen						X	X	X	X	X	X	X	X	X
Abfrage von Schüben**	X	X	X	X		X	X	X	X	X	X	X	X	X
Abfrage Krankenhaus-aufenthalte	X	X	X	X		X	X	X	X	X	X	X	X	X
Medikamenten-Compliance								X			X			X
Ausgabe von Medikamenten				X				X			X			
Rückgabe von Medikamenten								X			X			X
Schubdokumentation						Je nach Bedarf								
Unterbrechung der Studienmedikamenten						Je nach Bedarf								
Ende der Studie	je nach Bedarf													X*

1)Demographische Daten: Geburtsdatum, Geschlecht, ethnische Gruppe; 2)Vitalparameter: Herzfrequenz, systol./diastol. Blutdruck

3)Erweitertes Routinelabor: **EDTA** 2,7ml (Diff.-BB), **Heparin** 2,6ml (CK, Natrium, Kalium, Chlorid, Calcium, Retentionswerte, Cholesterin, Triglyceride, HDL-Chol., LDL-Chol., ASAT (SGOT), ALAT (SGPT), γGT, Bilirubin); **Citrat-Plasma** 5ml (Gerinnungsparameter); **Urinanalyse** (Eiweiß, Glukose, Sediments, Leukozyten, Erythrozyten, bei Visit –3 Schwangerschaftstest). $\Sigma_{vollblut}$ **= 10,3ml**

4)Großes Routinelabor: **Heparin** 2,6ml (CK, Natrium, Kalium, Retentionswerte, ASAT, ALAT). $\Sigma_{vollblut}$ **= 2,6ml**

5)Kleines Routinelabor: **Heparin** 2,6ml (CK, ASAT, ALAT). $\Sigma_{vollblut}$ **= 2,6ml**

6)Immunologie: **EDTA** 2,7ml (Quantifizierung von Regulatorischen T-Zellen und Expression von HLA-DR); **Heparin** 10ml (Durchflußzytometrie: intrazelluäres IL4, IL10, IFNγ, TNF), 30ml (Assays für Proliferationsmessungen und zur Expression von Zell-Zyklus- und zytokin-regulatorischen Molekülen, wie CDK4, $p27^{kip1}$, STAT1/4/6); **PaxGene** 2,5ml (Genexpression von Transkriptionsfaktoren, wie z.B. CIITA, Transaktivator von MHC Klasse II); **Serum** 5,5ml (für HMGCoA Reduktase Aktivität).
$\Sigma_{vollblut}$ **= 50,7ml**

7)MRT Routine: T2-, T1-, T1+ Kontrastmittel-Untersuchung

8)MRT Erweitert: Routine + Perfusions- + Diffusionsuntersuchung

9)Schwangerschaftstest im Serum (5,5ml für β-HCG, für diesen Untersuchungstermin kein Serum für die immunologischen Untersuchungen)

*Untersuchungen, die für den Monat 9 geplant sind, werden durchgeführt, wenn der/die Patient/in die Studie frühzeitig beendet.

**Wenn ein/e Patient/in einen Schub erfährt, dann müssen jeweils Ein- und Ausschlusskriterien überprüft werden.

3.14. Labor

Die Laboruntersuchungen im Detail sind in 3.13. aufgeführt. Während der Studie werden Serumchemie, Hämatologie und Urinanalyse als Sicherheitsmaßnahme durchgeführt. Bei der Rekrutierung (Monat -3) (Urintest) und vor Behandlungsbeginn (Monat 0) (beta-HCG Blutprobe) wird für Patientinnen ein Schwangerschaftstest gemacht. Nicht-nüchterne Blut- und Urinproben werden genommen. Sollte die Serumchemie in den nicht-nüchternen Proben außerhalb der Normwerte liegen, werden neue Untersuchungen mit nüchternen Proben gemacht. Jegliche Wiederholung von Laboruntersuchungen wird dokumentiert.
Die unter Immunologie aufgeführten Untersuchungen dienen der Erarbeitung des Wirkmechanismus von Atorvastatin in MS-Patienten.

3.15. Der/Die Untersucher/in

Der/Die Untersucher/in (ein/e Neurologe/in oder eine andere Person, die schon mit der Versorgung von MS-Patienten viel Erfahrung hat) ist für die gesamte medizinische Versorgung der/s Patientin/en, einschließlich die Handhabung und Einschätzung des Schwierigkeitsgrads der Schübe, verantwortlich. Andere wichtige Aufgaben sind: die Durchführung von ärztlichen Untersuchungen, die Bewertung der subjektiven Befunde der Patienten, die Verabreichung und das Monitoring des Testmedikaments sowie die Bewertung und Handhabung eines unerwünschten und unerwarteten Ereignisses („Adverse Event“=AE). Der/Die Untersucher/in führt auch die standardisierte neurologische Untersuchung durch und dokumentiert die neurologischen Symptome und Befunde je nach EDSS. Eine Studienassistent/in kann den MSFC durchführen.
Um die Konsistenz in den Patientendaten zu ermöglichen, soll möglichst der/die gleiche Arzt/Ärztin die/den gleiche/n Patientin/en während der ganzen Studie beobachten.
Mündliche Kommunikationen zwischen Studienpatienten über ihre Erfahrungen während der Studie sollen vermieden werden.

4. Auswertung der Daten

Die gesamte statistische Auswertung, einschließlich Dokumentation, Datenmanagement, Datenverarbeitung erfolgt unter Supervision des Institutes für Medizinische Biometrie der Charité, Prof. Dr. Klaus-D. Wernecke, Campus Virchow-Klinikum, in Zusammenarbeit mit dem Koordinationszentrum für klinische Studien (KKS) der Charité, Medizinische Fakultät der Humboldt-Universität zu Berlin.

4.1. Studiendesign

Es handelt sich um eine monozentrische, offene, prospektive, kontrollierte Studie im Cross-Over-Design (baseline-to-treatment) der Phase II.

4.2. Zielparameter

Primäre Zielparameter

Anzahl an Gd-anreichernden Läsionen auf dem T1-gewichteten Scan

Sekundäre Zielparameter

- Anzahl an neuen Läsionen auf dem T2-gewichteten Scan
- Anzahl an größer werdende Läsionen auf dem T2 -gewichteten Scan
- Anzahl an Läsionen auf dem T2-gewichteten Scan
- Volumen der Gd-anreichernden Läsionen auf dem T1-gewichteten Scan
- Volumen der hyperintensiven Läsionen auf dem T2-gewichteten Scan
- Scan-Aktivität (aktiv=neue aktive Läsionen, inaktiv=keine solche)

Weitere sekundäre Zielparameter

- Anzahl der Schübe
- Schubfreiheit unter Behandlung
- Veränderungen des MSFC
- Veränderungen des EDSS
- Veränderungen immunologischer Parameter

Um eine zuverlässige Analyse zu versichern, dürfen die MRT-Untersuchungen für diese Studie nicht durchgeführt werden, solange ein/e Patient/in intravenöse Steroidtherapie für einen Schub bekommt und bis 7 Tage nach Beendigung dieser Therapie. Sollte die Steroidtherapie 7 Tage vor dem nächsten MRT Termin nicht vollendet werden, dann muss das MRT früher gemacht und direkt vor der Steroidbehandlung durchgeführt werden; das darauf folgende MRT soll beim nächsten festgelegten Termin durchgeführt werden. Wenn die intravenösen Steroide nicht innerhalb der 7 Tage vor dem nächsten festgelegten Termin gegeben werden, dann kann das MRT wie geplant durchgeführt werden. Steroidtherapie für Schübe muss erfasst und dokumentiert werden.

4.3. Statistische Hypothesen

Die Studie soll primär klären, ob eine 9-monatige Behandlung mit Atorvastatintabletten mit 80 mg oral/die gegenüber einer 3-monatigen Baseline eine Reduktion der Anzahl der Gd-aufnehmenden Läsionen im MRT ergibt. Zur Sicherheit (der gewählte primäre Endpunkt könnte eine schiefe Verteilung aufweisen) gehen wir von vornherein von einer Prüfung mit einem nichtparametrischen Test aus und testen demzufolge die Hypothesen:

Nullhypothese H0: $F(B) = F(T)$ bzw. $P(T \leq B) = 1/2$

(Die Verteilungen von Baseline F(B) und Treatment F(T) stimmen überein bzw. die Wahrscheinlichkeit, dass die dem Treatment entsprechende Zufallsvariable T kleinere Werte annimmt als die für Baseline B, ist 0.5, also kein Unterschied)

Alternativhypothese (zweiseitig formuliert): $F(B) \neq F(T)$ bzw. $P(T \leq B) \neq 1/2$

(Die Verteilungen von Baseline F(B) und Treatment F(T) stimmen nicht überein bzw. die dem Treatment entsprechende Zufallsvariable T nimmt kleinere Werte [($P(T \leq B) > 1/2$] oder größere Werte [$P(T \leq B) < 1/2$] an als die entsprechende Baseline-Variable B).

4.4. Klinisch relevante Unterschiede zwischen Baseline und Behandlung

Der bereits in Abschnitt 1.2 erwähnte Beitrag auf der diesjährigen Tagung der amerikanischen neurologischen Gesellschaft, bereffend 45 MS-Patienten, die 6 Monate lang mit Simvastatin, einem dem Prüfpräparat vergleichbarem Statin, behandelt wurden, bildet die Grundlage der biometrischen Planung der hier vorliegenden Untersuchung. In der zitierten Studie wurden bei

einer 3-monatigen Periode vor Therapiebeginn und 6-monatiger Behandlungsphase die folgenden mittleren Werte der Gd-aufnehmenden Läsionen im MRT (jeweils arithmetischer Mittelwert ± Standardabweichung) erreicht:

vor Behandlung: 2.35 ± 1.94
nach Behandlung: 1.31 ± 1.30 (p=0.0001) (Vollmer et al., 2003).

Diese Werte bilden die Grundlage der Fallzahlplanung.

4.5. Statistische Analyse der Zielparameter

Alle Zielgrößen (primäre und sekundäre) werden zunächst mit Methoden der exploratorischen Datenanalyse untersucht und deskriptiv ausgewertet (Wernecke, 1995). Als primärer Endpunkt wurde die Anzahl an Gd-aufnehmenden Läsionen im MRT über den gesamten Behandlungszeitraum im Vergleich mit der Baseline gewählt. Eine eventuelle Erniedrigung der Anzahl im Behandlungszeitraum gegenüber der Baseline wird mit einem Zweistichprobentest für abhängige Stichproben nichtparametrisch mit dem Wilcoxon-Rang-Test (Büning und Trenkler, 1994) analysiert. Der gewählte primäre Endpunkt spricht wegen einer zu erwartenden schiefen Verteilung für einen nichtparametrischen Test, zumal mit nicht sehr großen Stichprobenumfängen zu rechnen ist. In Übereinstimmung damit sind auch die Hypothesen in Abschnitt 4.3. für einen nichtparametrischen Test formuliert. Der entsprechende Test wird mit einem Fehler 1.Art $\alpha = 5\%$ bei zweiseitiger Prüfung durchgeführt. Zur Prüfung der in 4.3. aufgestellten Hypothesen werden dabei die Patienten aus beiden Strata (vorbehandelt und nicht-vorbehandelt) zusammengefasst, d.h. der Test des primären Endpunktes erfolgt zunächst ohne Berücksichtigung der Vorbehandlung. Eine Überprüfung unter Einbeziehung der vorgesehenen Stratifizierung erfolgt im nächsten Schritt ebenfalls über ein nichtparametrisches Modell (nichtparametrische multivariate Varianzanalyse für wiederholte Messungen in einem zweifaktoriellen Design, vgl. Brunner et al., 2002) in einer zusätzlichen komplexen Analyse des primären Endpunktes der Art:

Stratum		Cross-Over	
		Baseline	Treatment
Vorbehandelt	Pat.1<br>…<br>Pat. n1	X_{V11}<br>…<br>X_{Vn11}	X_{V12}<br>...<br>X_{Vn12}
Nicht vorbehandelt	Pat.1<br>...<br>Pat. n2	X_{NV11}<br><br>X_{NVn21}	X_{NV12}<br><br>X_{NVn22}

(hierbei bedeuten die Symbole X_{Vij}, X_{NVij} die Anzahl an Gd-aufnehmenden Läsionen für den jeweils vorbehandelten (V) bzw. nicht-vorbehandelten (NV) i-ten Patienten (V: i = 1, 2,..., n1; NV: i = 1, 2,..., n2) im j-ten Cross-Over (j = 1, 2). Mit diesem Modell kann sowohl der Effekt der Behandlung in jedem Stratum einzeln als auch Wechselwirkungen zwischen Behandlung und Stratum, also ob der Effekt in einem speziellen Stratum besonders ausgeprägt ist, getestet werden. Diese Tests wie auch die nachfolgende Prüfung der sekundären Endpunkte erfolgen allerdings explorativ, d.h. die sich dabei eventuell ergebenden p-Werte < 5% (bei allgemein zweiseitigem Testen) bedeuten keine Konfirmation vorab formulierter Hypothesen (und werden deshalb auch nicht multipel adjustiert), sondern stellen lediglich interessante Resultate dar, die gegebenenfalls in nachfolgenden prospektiven klinischen Studien statistisch korrekt

zu prüfen wären. Die sekundäre Endpunkte werden je nach Skalierung und Verteilungstyp der Beobachtungswerte mit parametrischen Tests oder nichtparametrischen Pendants ausgewertet (Hartung, 1993), wobei im Falle von geringen Stichprobenumfängen, Unbalanziertheit oder nur spärlich besetzten Kontingenztafeln exakte Tests zur Prüfung verwendet werden.
Die Auswertung von objektivierbaren MRT-Parametern für den primären Endpunkt, welche über einen Zeitraum von 9 Monaten sensitiver als die klinische Untersuchung anzusehen ist, muss aber auf jeden Fall verblindet erfolgen.

4.6. Fallzahlplanung

Ausgangspunkt der Fallzahlplanung sind die in Abschnitt 4.3. aufgestellten Hypothesen und die aus einer Vorläuferstudie in Abschnitt 4.4. angeführten Eckdaten. Damit ergibt sich bei Prüfung mit dem nichtparametrischen Test nach Wilcoxon (Mann-Whitney) für unabhängige Stichproben sowie den Parametern α = 5% (zweiseitiger Test), β = 20%, d.h. Power von 80% und den Eckdaten aus 4.4. ein Stichprobenumfang von n = 48. Diese Fallzahl stellt den „worst case" dar und ist im Fall von gepaarten Stichproben (Cross-Over) infolge der korrelierten Beobachtungen sicher noch zu unterschreiten. Prüft man über ein Design mit zwei Messwiederholungen (baseline, Treatment) und einer Korrelation zwischen den Wiederholungen von 0.5, nun aber parametrisch (Fallzahlschätzungen für dieses Design im nichtparametrischen Fall existieren zur Zeit noch nicht), so erhält man n = 42 Patienten als Fallzahl. Mit einer geschätzten Drop-Out-Rate von 10% kann man also sicher von insgesamt von n = 50 Patienten ausgehen (vgl. auch die folgende Abbildung für den nichtparametrischen Test).

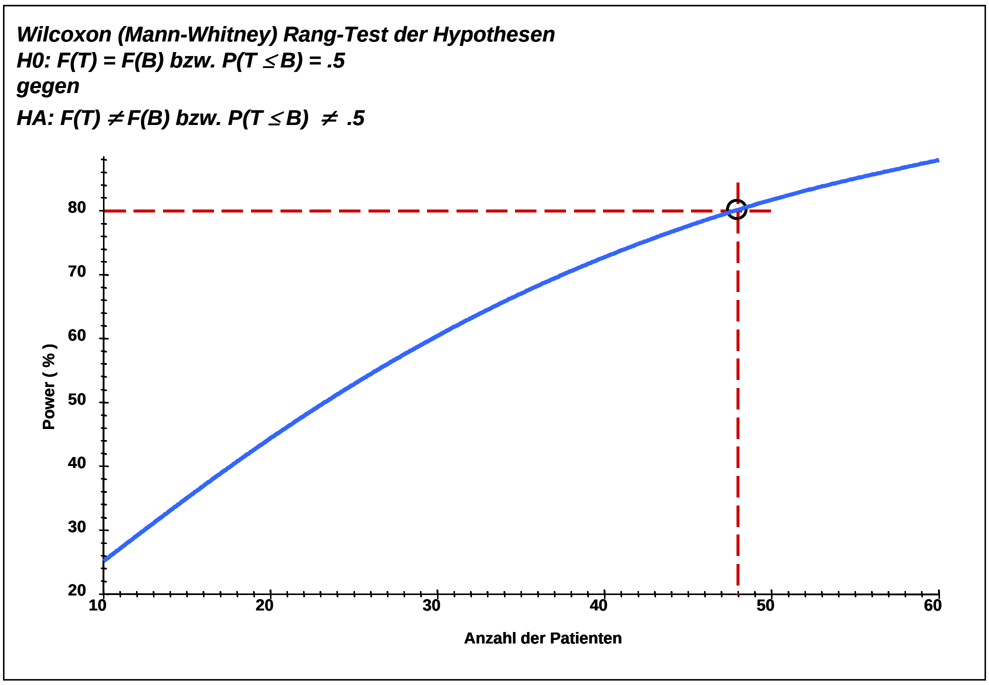

Abbildung: Zusammenhang zwischen Fallzahl und Power

4.7. Definition von Auswertungskollektiven

Die Analyse bezüglich des Hauptzielkriteriums (primärer Endpunkt) erfolgt zunächst unter Berücksichtigung aller Patienten, die dem Studienregime strikt gefolgt waren (*per protocol*).

In die *intention-to-treat*-Analyse werden alle Patienten einbezogen, die mit der Studientherapie behandelt wurden. Die Ergebnisse beider Analysen werden verglichen, eventuelle wesentliche Abweichungen sind zu diskutieren.

Statistische Referenzen

- Brunner, E., S. Domhoff and F. Langer (2002): Nonparametric Analyis of Longitudinal Data in Factorial Experiments, Wiley, New York
- Büning, H. und G.Trenkler (1994): Nichtparametrische statistische Methoden, Walter de Gruyter, Berlin, New York
- Hartung K., Elpelt B.(1993): Statistik, Oldenbourg Verlag München, 9.Auflage
- Vollmer T, Durkalski V, Tyor W, Corboy J, Preiningerova J, Markovic-Plese S et al. An open-label, single arm study of simvastatin as a therapy for multiple sclerosis (MS). AAN 2003
- Wernecke, K.-D. (1995): Angewandte Statistik für die Praxis, Addison-Wesley, Bonn

5. Dokumentation

5.1. Datenerhebung / Dokumentationsbögen/Datenverarbeitung

Die erhobenen Befunde, Messergebnisse, Begleiterscheinungen und alle gemäß Prüfplan erhobenen Daten werden in die Prüfbögen eingetragen. Jeder einzelne Prüfbogen muss vom Arzt mit dem Datum und seiner Unterschrift versehen werden. Korrekturen sind wie folgt vorzunehmen: Der falsche Eintrag wird mit einer einfachen Linie durchgestrichen, die korrekte Information daneben eingetragen und vom Prüfarzt mit Datum paraphiert und ggf. mit Angabe des Grundes der Korrektur versehen. Datenfelder, die wegen fehlender Information nicht ausgefüllt werden können, sind zu kommentieren. Die Bögen sind zeitnah auszufüllen und anschließend vom Prüfarzt zu kontrollieren, mit Datum zu unterschreiben und der Studienzentrale zuzuleiten.

Die validierten Daten werden in einer geeigneten Datenbank abgelegt. Am Studienende wird nach Eingabe aller Eintragungen die Datenbank geschlossen. Dieser Vorgang wird dokumentiert. Für die statistische Auswertung wird eine geeignete Software verwendet (SAS, SPSS oder S-PLUS), die das Institut für Medizinische Biometrie der Charité zur Verfügung stellt.

5.2. Demographische Informationen

Folgende Daten werden bei der Rekrutierung erfasst:

- demographische Daten:
  Dokumentation des Geburtsdatums, des Geschlechts und der ethnischen Gruppe

- Regelanamnese:
  Die Anamnese der Menstruation wird dokumentiert

- Schwangerschaft

- MS-Diagnose

Die MS-Diagnose muss nach McDonald et al. (2001) bestätigt werden:
Mindestens zwei Schübe; objektive klinische Beweise für mindestens zwei Läsionen[a]

ODER

Mindestens zwei Schübe; objektive klinische Hinweise für eine Läsion und eine im MRT nachgewiesene räumliche Dissemination[b], oder für mindestens zwei MS-typische Läsionen im MRT *plus* positivem Liquorbefund[c].

ODER

Ein Schub; objektive klinische Hinweise für mindestens zwei Läsionen und eine im MRT nachgewiesene zeitliche Dissemination[d]

ODER

Ein Schub; objektive klinische Hinweise für eine Läsion (monosymptomatische Präsentation; klinisch isoliertes Syndrom) und für eine im MRT nachgewiesene räumliche Dissemination[b], oder mindestens zwei Läsionen im MRT *plus* positivem Liquorbefund[c] und im MRT nachgewiesene zeitliche Dissemination[d]

[a]Keine weiteren Tests sind erforderlich; sollten die Untersuchungen [MRT, Liquor] durchgeführt werden und negativ sein, dann muss man extrem vorsichtig mit der Diagnose von MS sein. Alternative Diagnosen müssen berücksichtigt werden. Es darf keine bessere Erklärung für das klinische Bild geben.

[b]Der MRT Nachweis von räumlicher Dissemination muss drei von vier der folgenden Kriterien erfüllen:

1. Eine Gd-anreichernde Läsion oder neun T2-hyperintensive Läsionen bei Fehlen von Gd-anreichernden Läsionen.
2. Mindestens eine infratentorielle Läsion
3. Mindestens eine juxtakortikale Läsion
4. Mindestens drei periventrikuläre Läsionen.

[c]Positiver Liquor mit oligoklonalen Banden (vorzugsweise in der isoelektrischen Fokussierung) (nicht im Serum) oder mit einem erhöhten IgG-Index.

[b]Der MRT Nachweis zeitlicher Dissemination muss diese Kriterien erfüllen:
1. Wenn der erste Scan mindestens 3 Monaten nach dem Beginn des klinischen Ereignisses stattfindet, dann ist das Vorkommen einer Gd-anreichernden Läsion ausreichend, um eine zeitliche Dissemination festzustellen, vorausgesetzt, dass die Läsion nicht an der Stelle liegt, wo das ursprüngliche klinische Ereignis zu erwarten ist. Falls sich keine Gd-anreichernde Läsion in diesem Scan findet, erfüllen eine neue T2- oder Gd-anreichernde Läsion in einem Follow-up Scan wiederum mindestens 3 Monate später das Kriterium für eine zeitliche Dissemination.
2. Wenn der erste Scan nach weniger als 3 Monaten nach dem Beginn des klinischen Ereignisses stattfindet, dann ist ein zweiter Scan nach mindestens 3 Monaten nach dem klinischen Ereignis ausreichend, der eine neue Gd-anreichernde Läsion zeigt, um eine zeitliche Dissemination festzustellen. Zeigt sich diese nicht, dann ist ein weiterer Scan mindestens 3 Monate nach dem ersten Scan notwendig, welcher dann eine neue T2-Läsion oder Gd-anreichernde Läsion zeigt.

- Anamnese der MS

- Anamnese von Schüben:
  Dokumentation der Gesamtzahl an Schüben seit dem Beginn und während der vergangenen 12 und 24 Monate. Detaillierte Dokumentation des neuesten Schub.

- Medizinische und chirurgische Anamnese:
  Die medizinische Anamnese der folgenden Organsysteme wird für die relevanten Erkrankungen geprüft:

Haut, Augen, Ohren, Nase, Kopf und Hals (einschließlich der Schilddrüse), Lungen, Herz, Brüste, Bauch (Magen-Darm, Stuhlgewohnheiten; und Leber, Nieren, Milz u.a.), Lymphknoten, Skelettmuskulatur (einschließlich der Extremitäten und der Wirbelsäule), Urogenitalsystem, gynäkologische Organe und Rektum. Anamnese von Allergien wird erfasst.
Darüber hinaus wird die Anamnese von Drogen- oder Alkoholmissbrauch und psychiatrischen Störungen erhoben. Patienten mit extremer Depression oder einem Selbstmordversuch in dessen/deren medizinischen Vorgeschichte (oder mit aktueller Suizidalität) dürfen für diese Studie nicht selektiert werden.

- Medikamentenanamnese:
  Bei Rekrutierung Dokumentation der Medikamente, die in den vorangehenden 6 Monaten genommen wurden (generischer Name, Indikation, Dosierung, Frequenz und Art der Verabreichung, und das Beginn-/Enddatum). Im Verlauf muss jegliches neue Medikament geprüft und dokumentiert werden.

- Überprüfung der Ein-/Ausschlusskriterien:
  Bestätigung in der Studienakte, dass sämtliche Ein-/Ausschlusskriterien für den/die Patient/in erfüllt wurden.

- MRT-Charakteristika

Die Detail-Dokumentation der darauffolgenden Untersuchungstermine ist der Studienakte zu entnehmen. Zu den MRT-Untersuchungen wird jeweils nur dokumentiert, ob Gd-anreichernde Läsionen zu sehen sind oder nicht und ob eine Zunahme an T2-Läsionen zu sehen ist oder nicht. Im übrigen wird die Verschlüsselung der mehrfach gespeicherten Scans in der Studienakte notiert. Die Auswertung der Daten erfolgt für alle Daten zu einem Zeitpunkt und geblindet.

5.3. Sicherheit

Unerwünschtes Ereignis (Adverse Event = AE)

Ein AE ist jedes unerwünschte medizinische Ereignis, das bei einem Teilnehmer an einer klinischen Prüfung nach Verabreichung eines Arzneimittels auftritt und das nicht unbedingt in ursächlichem Zusammenhang mit dieser Behandlung steht. Ein unerwünschtes Ereignis kann

- jede ungünstige/unbeabsichtigte Reaktion des Körpers (z.B. anormaler Laborbefund)
- jedes Symptom oder
- jede vorübergehend mit der Verabreichung eines Arzneimittels (hier Prüfpräparat) einhergehende Erkrankung sein, ob diese nun mit dem Prüfpräparat in Zusammenhang steht oder nicht
- jede Verschlechterung einer bestehenden Erkrankung z.B. in der Intensität/Rezidivhäufigkeit sein

Schwerwiegendes unerwünschtes Ereignis (Serious Adverse Event=SAE)

Als schwerwiegendes unerwünschtes Ereignis (unabhängig von einem möglichen Kausalzusammenhang mit der Prüfmedikation) wird jedes ungünstige medizinische Ereignis, wenn

- es zum Tod führt,
- es lebensbedrohend ist,
- es zu Arbeitsunfähigkeit oder einer Behinderung führt,
- es eine stationäre Behandlung oder die Verlängerung einer stationären Behandlung zur Folge hat,
- es zur teratogenen Schädigung (z.B. Schädigung des Erbgutes durch Mutationen) führt,
- ein maligner/neoplastischer Prozess diagnostiziert wird

Zu jeder Zeit der Studie muss das Vorkommen von AE besonders beachtet werden. So muss der/die Patient/in von dem/r Untersucher/in sowohl während als auch nach der Untersuchung genau beobachtet werden.

Alle AE müssen im Detail dokumentiert werden. Folgende Informationen sind erforderlich:

- Erkrankung, Zeichen oder Symptome, die ein AE charakterisieren
- Datum und Uhrzeit
- Schwerwiegendes AE (=SAE) (ja, nein)
- Intensität
- Muster (bei jeder Verabreichung des Medikaments, unterbrochen, kontinuierlich, u.s.w.)

(die folgende Einzelheiten werden dann notiert, wenn das AE aufgehört hat, relevante Veränderungen passieren oder spätestens bis Ende der Studie)

- die am meisten relevante Veränderung der Studienmedikation wegen des AE (Medikament abgesetzt, Dosierung reduziert, Dosierung unverändert, keine Angabe), und weitere Details, wenn angebracht.
- spezifische medikamentöse Behandlung des AE
- spezifische nicht medikamentöse Behandlung des AE
- das Ergebnis/die Folge des AE (vollständig zurückgebildet, in Rückbildung, nicht vollständig zurückgebildet, tödlich, nicht bekannt)
- AE hält an mit Residualsymptomatik

Immer wenn eine Veränderung der Intensität (zu einer anderen Kategorie, d.h. gering, mittel, stark, siehe unten) eines spezifischen AE vorkommt, muss eine zusätzliche AE-Seite ausführlich ausgefüllt werden.

Intensität

gering:	Die Intensität des Ereignisses wird als mild angesehen, wenn die mögliche Variationsbreite der Intensität dieses Ereignisses berücksichtigt wird.<br>Normalerweise wird ein AE als mild angesehen, wenn ein/e Patient/in Zeichen und Symptome verspürt, diese aber gut toleriert.
mittel:	s.o.

	Normalerweise wird ein AE als moderat angesehen, wenn die Zeichen und Symptome eines/r Patienten/in die üblichen Aktivitäten einschränken, aber nicht verhindern.
stark:	s.o.<br>Normalerweise wird ein AE als schwerwiegend bezeichnet, wenn der/die Patient/in die üblichen Aktivitäten nicht mehr durchführen kann.

Schwerwiegende bzw. unerwartete unerwünschte Ereignisse, die während der Studie auftreten und die Sicherheit der Patienten oder die Durchführung der Studie beeinträchtigen könnten, müssen der Ethikkommission gemeldet werden. Gemäß GCP sollte der zuständigen Ethikkommission nach Abschluss der Studie ein Abschlussbericht der Studie nach den lokalen Begebenheiten vorgelegt werden. Bei vorzeitigem Abbruch einer Studie ist dies ebenfalls der zuständigen Ethikkommission mitzuteilen.
Die Meldungen an das Bundesamt für Arzneimittel und Medizinprodukte übernimmt das KKS der Charité.

5.4. Qualtitätssicherung

Der Sponsor-Investigator beauftragt das KKS Charité mit dem Monitoring. Der Monitor besucht das Prüfzentrum nach Einschluss des ersten Patienten, im Verlauf der Studie sowie nach Beendigung der Behandlung des letzten Studienpatienten.

Der Monitor hat die Verantwortung gemeinsam mit dem Prüfarzt die Durchführung der Studie gemäß Studienprotokoll zu verifizieren. Die Wahrung der Vertraulichkeit der Studiendokumente einschließlich der Patientendaten ist dabei zu gewährleisten.

Ein Teil der Befunde wird direkt in der Studienakte dokumentiert und gelten somit als Quelldaten. Die übrigen Befunde werden einer Quelldatenkontrolle unterzogen.

____

Datum und Unterschrift der verantwortlichen Prüfarztes
(Antragssteller)

<u>Anlagen</u>
Fachinformation Sortis® (Atorvastatin)
Patienteninformation
Einwilligungserklärung
CV des Leiters der Klinischen Prüfung
